# Induction of Human T-cell and Cytokine Responses Following Vaccination with a Novel Influenza Vaccine

**DOI:** 10.1038/s41598-018-36703-7

**Published:** 2018-12-20

**Authors:** David A. G. Skibinski, Leigh Ann Jones, Yuan O. Zhu, Lin Wu Xue, Bijin Au, Bernett Lee, Ahmad Nazri Mohamed Naim, Audrey Lee, Nivashini Kaliaperumal, Jenny G. H. Low, Lawrence S. Lee, Michael Poidinger, Philippe Saudan, Martin Bachmann, Eng Eong Ooi, Brendon J. Hanson, Veronica Novotny-Diermayr, Alex Matter, Anna-Marie Fairhurst, Martin L. Hibberd, John E. Connolly

**Affiliations:** 1 Institute of Molecular and Cell Biology, 61 Biopolis Drive, Proteos Bldg, Singapore, 138673 Singapore; 2 A*STAR Program in Translational Research on Infectious Disease, Agency for Science, Technology and Research, Singapore, Singapore; 3 Genome Institute of Singapore, Agency for Science, Technology and Research, Singapore, Singapore; 4 Singapore Immunology Network (SIgN), 8A Biomedical Grove, Immunos Bldg, Singapore, 138648 Singapore; 5 SingHealth Investigational Medicine Unit, Singapore General Hospital, Block 7, Outram Rd., Singapore, 169610 Singapore; 6 Singapore General Hospital, 20 College Road, Singapore, 169856 Singapore; 7 Clinical Trials Research Unit, Changi General Hospital, 2 Simei St. 3, Singapore, 529889 Singapore; 8 National University of Singapore, Department of Medicine, 1E Kent Ridge Road, Singapore, 119228 Singapore; 9 Cytos Biotechnology AG, Wagistr. 25, Zürich-Schlieren, CH-8952 Switzerland; 10 Inselspital, RIA, Immunologie, Sahlihaus 2, CH-3010 Bern, Switzerland; 11 Duke-NUS Medical School, Program in Emerging Infectious Diseases, 8 College Rd., Singapore, 169857 Singapore; 12 DSO National Laboratories, Bio-Defense Therapeutics Lab, 27 Medical Drive, Singapore, 117510 Singapore; 13 D3 (Drug Discovery and Development), 31 Biopolis Way, #01-02a Nanos, Singapore, 138669 Singapore; 14 Institute of Biomedical Studies, Baylor University, Waco, Texas 76712 USA

## Abstract

Cell mediated immunity plays a vital role in defense against influenza infection in humans. Less is known about the role of vaccine-induced cell mediated immunity and the cytokine responses elicited. We measured CD4^+^ and CD8^+^ T-cell reactivity in human subjects following vaccination with licensed trivalent influenza vaccine and a novel virus-like particle based vaccine. We detected influenza-specific CD4^+^ T-cell responses following vaccination with the licensed trivalent influenza vaccine and found that these correlated with antibody measurements. Administration of the novel virus-like particle based vaccine elicited influenza-specific CD4^+^ and CD8^+^ T-cell responses and the induction of the cytokines IFN-γ, IL-17A, IL17F, IL-5, IL-13, IL-9, IL-10 and IL-21. Pre-existing cytokine responses influenced the profile of the cytokine response elicited by vaccination. In a subset of individuals the VLP vaccine changed pre-vaccination production of type 2 cytokines such as IL-5 and IL-13 to a post-vaccination type 1 cytokine signature characterized by IFN-γ. A transcriptional signature to vaccination was found to correlate with antibody titer, IFN-γ production by T-cells and expression of a putative RNA helicase, DDX17, on the surface of immune cells.

## Introduction

The most established correlate of protection against influenza infection are antibodies targeting influenza virus envelope glycoprotein haemagglutinin (HA)^1^. However numerous clinical studies have demonstrated an important role for T-cells in driving protection. The number of influenza-specific interferon-γ (IFN-γ) producing CD4^+^ T-cells negatively correlate with the development of disease in antibody-naive healthy volunteers following influenza challenge^2^. Another study reported that the frequency of influenza-specific IFN-γ producing CD8^+^ T-cells positively correlated with less severe illness in a healthy adults following natural^3^.

Immune responses to influenza vaccination are characterized by antibody levels with licensure criteria dependent on haemagglutinin inhibition (HAI) titers^4^. However, currently available vaccine regimens, fail to confer protection to all individuals, particularly elderly subjects^5^. The current Trivalent Influenza Vaccine (TIV) is poor at eliciting CD4^+^ T-cell^6–15^ or CD8^+^ T-cell^11,16^ responses after vaccination, and much recent focus has been on finding an association between T-cell responses and influenza specific antibody responses^17–20^. Nayak *et al*.^19^ found that, in individuals vaccinated with the monovalent inactivated pandemic 2009 H1N1 vaccine, the expansion of influenza- CD4^+^ T-cells correlated with neutralizing antibody titers. Research with adjuvanted influenza vaccine, found that the presence of CD4^+^ T-cells specific for the vaccine were predictive of long term maintenance of protective antibody titers^18^. A follow up study revealed that influenza-specific ICOS^+^ IL-21^+^ CD4^+^ T-cells were associated with a rise in functional antibodies^20^. Together this work has made important contributions towards understanding the input of T-cells in driving humoral immunity following vaccination. However, less is known about the full range of T-cell responses that are induced across different T-cell subsets, and how pre-existing immunity can shape these responses post-vaccination.

Here, we utilized flow cytometry and multiplex-immunoassays to fully characterize T-cell responses and the spectrum of cytokine responses in healthy adults both before and after vaccination with TIV and a novel virus-like particle (VLP) based vaccine. We detected pre-existing influenza-specific responses in these individuals. Vaccination with both TIV and VLP enhanced the magnitude of T-cell responses without fundamentally altering the profile of cytokines produced. However, with the VLP vaccine, we identified a subset of individuals whose response shifted from a pre-existing T helper type 2 (Th2) cytokine profile bias towards a T helper type 1 (Th1) cytokine profile.

Finally, using whole blood transcriptional analysis, we determined that a putative RNA helicase, DDX17, is down regulated following vaccination with the virus-like particle based vaccine, and identified a transcriptional signature to vaccination which correlates with antibody titer, IFN-γ cytokine response and expression of DDX17 on the surface of circulating immune cells.

## Results

### CD4^+^ T-cell proliferation correlates with neutralizing antibody responses to trivalent influenza vaccination

**Figure 1:**
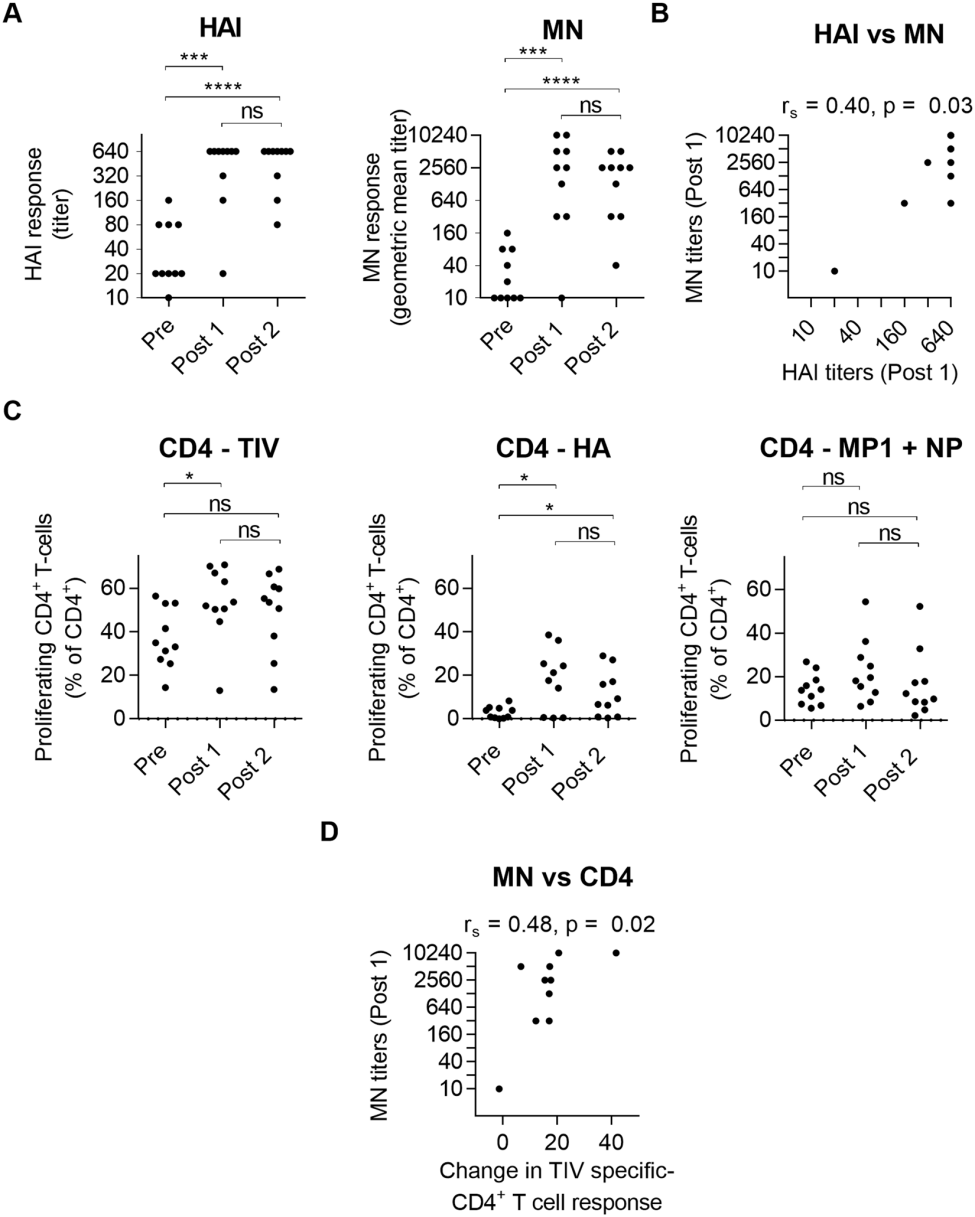
CD4^+^ T-cell responses predict neutralizing antibody responses to trivalent influenza vaccination. (**A**) HAI antibody responses (left) and MN antibody responses (right) against A/California/07/2009 following Fluarix vaccination. One way ANOVA. ****P < 0.0001, ***P < 0.001, Tukey’s test. ns, not significant. (**B**) Correlation between HAI and MN titers after one dose of vaccine. Spearman rank correlation test. n = 10, data points overlap. (**C**) Proliferating CD4^+^ T-cells in cultures of PBMCs isolated from subjects vaccinated with TIV. PBMCs were stimulated with the TIV vaccine and peptide pools specific for A/California/7/2009 HA, MP1 and NP proteins. Friedman test. *P < 0.05, Dunn’s test. ns, not significant. (**D**) Correlation between MN titer (Post 1) and the change in TIV-specific CD4^+^ T-cell response (post 1 response – baseline response). Spearman rank correlation test. Labels “Pre”, “Post1” and “Post2”, refer to Day 0, Day 21 and Day 42 post primary vaccination respectively.

Ten healthy adults aged 21–56 years received two doses of Trivalent Influenza Vaccine (TIV; A/California/7/2009 (H1N1), A/Perth/16/2009 (H3N2) and B/Brisbane/60/2008) twenty-one days apart. Geometric mean HAI titers to H1N1 increased from 35 before vaccination to 368 and 422 after one and two doses respectively (Fig. 1A). Seroconversion was observed in 9 subjects (90%) after one dose and in all subjects (100%) after two doses of the vaccine. Similar results were determined using the microneutralization (MN) assay, with a positive correlation (r_s_ = 0.40, p = 0.03) between the two assays (Fig. 1A,B).

To analyze the influenza specific T-cell responses to TIV vaccination, CFSE-labelled peripheral blood mononuclear cells (PBMCs) from vaccinated individuals were stimulated *in vitro* with the vaccine or with peptide pools specific for the HA and NP/MP1 influenza proteins. CD4^+^ T-cell proliferation was detected using CFSE dilution (Supplementary Fig. S1). There was a significant increase in proliferation following a single dose with either TIV or HA stimulation (Fig. 1C; Supplementary Table S1). HA-specific CD4 proliferative responses remained high following the second dose of vaccine. Proliferation of NP/MP1 specific CD4^+^ T-cells pre- and post-vaccination was equivalent despite NP and MP1 proteins being detectable in the vaccine using Mass Spectroscopy (Supplementary Table S1). There was no detection of influenza-specific CD8^+^ T-cell or B cell proliferation to TIV vaccination (Supplementary Fig. S2A and B). Stimulation with PMA and ionomycin did not increase response post vaccination (Supplementary Fig. S2C). After eight days *in vitro* stimulation proliferating TIV-specific CD4^+^ T-cells were predominantly positive for the T follicular helper (Tfh) markers ICOS and PD-1 yet, as previously described^20^, these influenza-specific T-cells were negative for CXCR5 (Supplementary Fig. S3). It is important to consider that the *in vitro* stimulation step has the potential to change the expression of those markers, and therefore it may not reflect their expression on these cells in blood.

As previously reported^19^ we found a correlation between the change in the TIV-specific CD4^+^ T-cell response and the MN titer (r2 = 0.48, p = 0.02) after one dose of the vaccine (Fig. 1D).

### The pre-existing influenza-specific cytokine profile is retained following TIV vaccination

**Figure 2:**
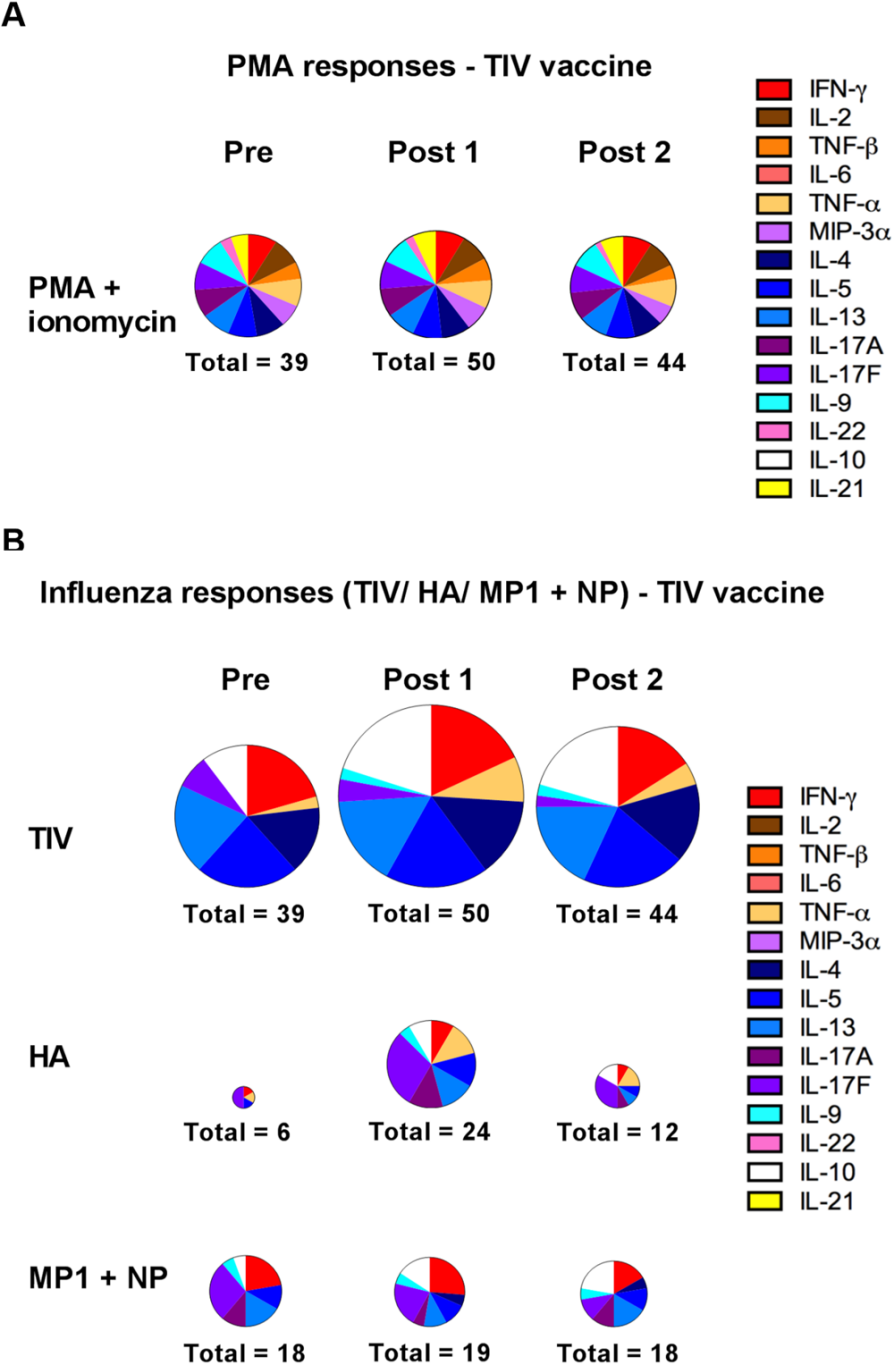
Quality of the pre-existing influenza-specific cytokine profile remains unchanged following TIV vaccination. (**A**,**B**) Distribution of positive cytokine responses from subjects before and after vaccination with trivalent influenza vaccine (n = 10). PBMCs were cultured with (**A**) PMA and ionomycin, and (**B**) the TIV vaccine and peptide pools specific for A/California/7/2009 HA, MP1 and NP proteins. Levels of the indicated cytokines were measured in the cell culture supernatants after eight days incubation. The size of the charts is proportional to the magnitude of the response (number of positive responses in all subjects for that visit is indicated for each chart; maximum of 150). Labels “Pre”, “Post1” and “Post2”, refer to Day 0, Day 21 and Day 42 post primary vaccination respectively.

To examine the quality of the cytokine response observed following TIV vaccination, TIV- and peptide- stimulated PBMC cultures were assayed for cytokine levels at day 8 post stimulation (Supplementary Tables S2 and S3). Of the 15 cytokines and chemokines tested only TIV-specific IL-10 levels (P < 0.01) were greater following vaccination (Supplementary Fig. S4). We found no correlation between cytokine response and MN titer (data not shown). Ideally, to look at the quality of the response, as opposed to the magnitude, we should look at the distribution of cytokine responses in relation to each other. However, comparing different cytokines is hampered by the fact that their relative levels are orders of magnitude apart. In an attempt to investigate this, we normalized the data by defining a positive response for each cytokine in each individual subject as being greater than two-standard deviations above the background for that analyte. As expected we found that positive cytokine responses were equally distributed following stimulation with PMA and ionomycin (Fig. 2A). Although, as described above, cytokine levels from TIV or HA stimulated PBMCs were largely unchanged there is a trend towards more individual positive responses following vaccination (Fig. 2B). The proportional distribution of these individual cytokine responses did not change following vaccination.

### Vaccination with gH1-Qbeta elicits influenza-specific CD4^+^ and CD8^+^ T-cell responses

**Figure 3:**
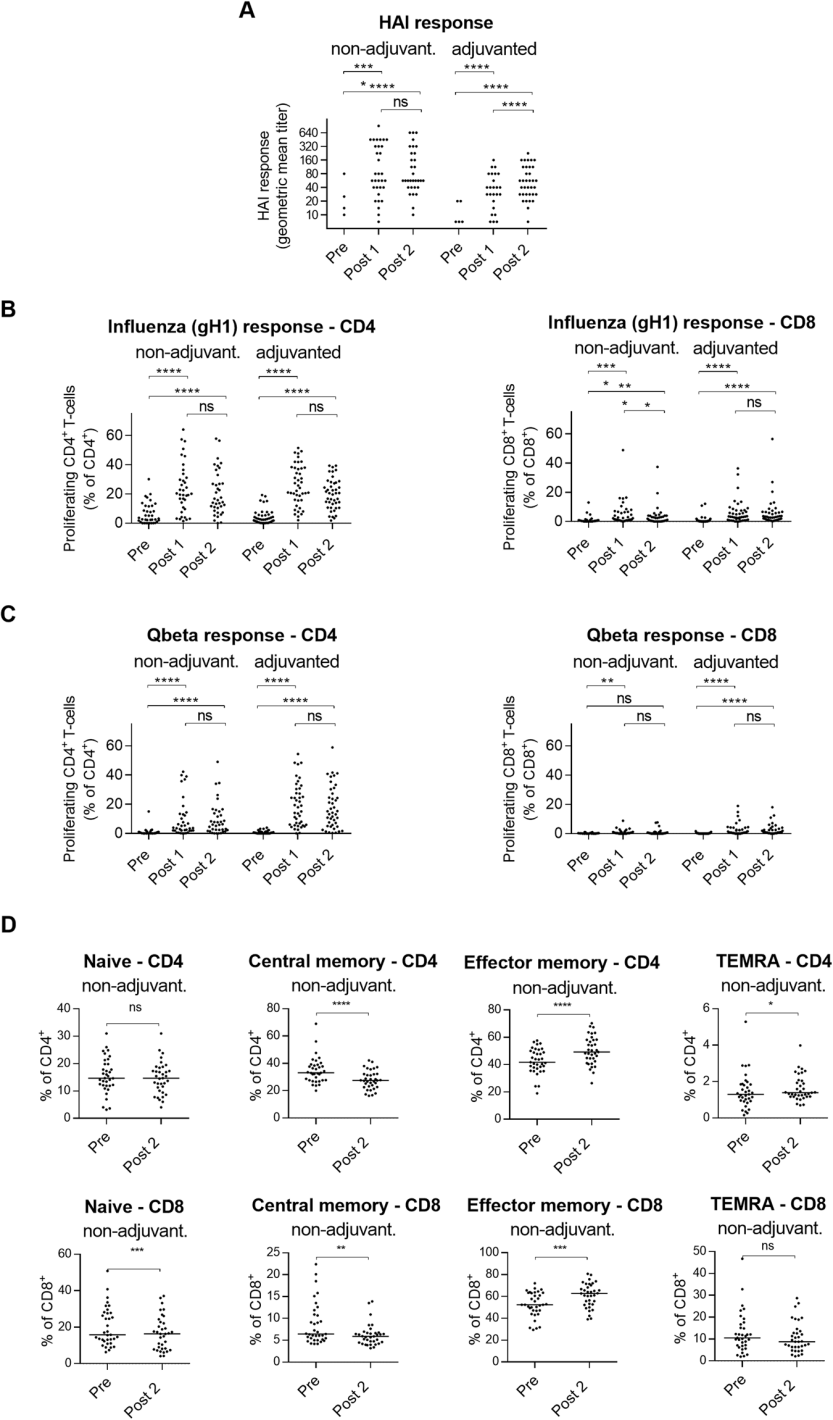
Vaccination with gH1-Qbeta vaccine elicits gH1-specific CD4+ T-cell proliferation. (**A**) HAI antibody responses against A/California/07/2009 following gH1-Qbeta vaccination. One way ANOVA. ****P < 0.0001, Tukey’s test. ns, not significant. (**B**) Proliferating gH1-specific CD4^+^ (left) and CD8^+^ (right) T-cells in cultures of PBMCs isolated from subjects vaccinated with non-adjuvanted and adjuvanted gH1-Qbeta vaccine. Friedman test. ****P < 0.0001, Dunn’s test. ns, not significant. (**C**) Proliferating Qbeta-specific CD4^+^ (left) and CD8^+^ (right) T-cells in cultures of PBMCs isolated from subjects vaccinated with non-adjuvanted and adjuvanted gH1-Qbeta vaccine. Friedman test. ****P < 0.0001, **P < 0.01 Dunn’s test. ns, not significant. (**D**) T-cell subsets present in PBMCs of cohort vaccinated with non-adjuvanted gH1-Qbeta. Wilcoxon test. ****P < 0.0001, ***P < 0.001, **P < 0.01 Dunn’s test. ns, not significant. Labels “Pre”, “Post1” and “Post2”, refer to Day 0, Day 21 and Day 42 post primary vaccination respectively.

The gH1-Qbeta vaccine is a novel pandemic-influenza vaccine produced by covalently linking the globular head domain of haemagglutinin (gH1) from A/California/07/09 produced in *E*. *coli* to VLPs from the bacteriophage Qbeta^21^. The vaccine has been evaluated in a Phase I clinical study where, following the regimen for the above TIV study, 84 healthy adults aged 21–64 years received two doses of the vaccine twenty-one days apart^22^. Subjects were randomized to receive alum-adjuvanted (n = 43) and non-adjuvanted vaccine (n = 41) with the latter formulation reaching the primary immunogenicity endpoint of 60% seroconversion (62.2% and 70.3% of individuals seroconverting after one and two doses of the respectively). The alum-adjuvanted formulation demonstrated inferior performance, failing its primary endpoint (seroconversion in only 25.5% and 51.2% of individuals after one and two doses respectively; Fig. 3A)^22^.

In an identical manner to the TIV challenge described above, CFSE-labelled PBMCs from the gH1-Qbeta vaccinated individuals were stimulated *in vitro* with peptide pools specific for gH1 and Qbeta. The gH1-Qbeta vaccine was found to elicit gH1-specific CD4^+^ and CD8^+^ T-cell responses regardless of adjuvant (Fig. 3B). Using the same gH1-specific peptide pool similar gH1-specific increases were not observed following TIV vaccination (Supplementary Fig. S5). As expected, there were no differences in PMA/ionomycin induced T cell activation pre- and post-vaccination (Supplementary Fig. S6).

Qbeta-specific challenge also resulted in increased CD4^+^ and CD8^+^ T-cell proliferation (Fig. 3C), and when directly compared, this increase was determined to be higher for the adjuvanted versus unadjuvanted vaccine following both one and two doses (p = 0.014 and p = 0.022 respectively). This enhancement of responses to Qbeta (the non-influenza specific component of the vaccine) could reflect the potential requirement of the adjuvant to elicit responses to antigens for which there is no pre-existing immunity. Contrary to our findings with TIV vaccine, we found no significant correlation between CD4^+^ or CD8^+^ T-cell proliferation and MN titers for either non-adjuvanted or adjuvanted groups (data not shown).

*Ex-vivo* analysis of PBMCs without simulation identified post vaccination contraction of the peripheral central memory pool of T-cells and an expansion of the effector memory pool (Figs 3D and S7).

### Vaccination with gH1-Qbeta elicits a poly-functional influenza-specific cytokine response

We then assessed the production of cytokines following exposure to gH1 peptides in gH1-Qbeta vaccinated individuals. We determined increases in IFN-γ, IL17A, IL-17F and IL-9 in both adjuvanted and non-adjuvanted groups (Table 1). Furthermore, the IFN-γ levels were found to correlate with CD4+ T-cell proliferation (Supplementary Fig. S8). In the adjuvanted group, gH1-specific increases in IL-5, IL-13 and IL-21 were also observed. We observed similar increases in gH1-specific responses in the TIV however, these were not significant (Supplementary Table S4).

**Table 1:** gH1-specific cytokine responses following vaccination with gH1-Qbeta.

Cytokine/Chemokine	Non-adjuvanted			Adjuvanted		
	Day 0	Day 21	Day 42	Day 0	Day 21	Day 42
Proinflammatory						
IFN-γ	34.18	**137.38**	**138.55**	37.76	**257.89**	**211.80**
IL-2	4.91	0.80	0.71	4.73	0.01	0.01
TNF-β (LTα)	0.01	0.01	0.01	0.01	0.01	0.01
IL-6	4996.66	15438.32	4752.26	5260.17	15465.00	4911.00
TNF-α	161.17	165.73	155.83	200.76	212.66	190.88
MIP-3α (CCL20)	143.70	174.80	84.49	141.99	140.32	100.57
Th2						
IL-4	0.01	0.01	0.01	0.01	0.01	0.01
IL-5	1.63	5.79	5.58	1.59	**5**.**28**	**5**.**39**
IL-13	34.41	73.18	58.48	31.72	90.44	68.04
Th17						
IL-17A	8.42	**30.64**	33.24	8.76	**20.88**	13.25
IL-17F	0.11	**0.40**	**0.25**	0.08	**0.40**	**0.28**
Th9						
IL-9	0.54	2.05	**2.48**	0.07	**3.25**	**5.39**
Th22						
IL-22	0.01	0.01	0.01	0.01	0.01	0.01
Treg						
IL-10	17.95	23.09	17.78	16.28	23.27	19.83
Tfh						
IL-21	0.01	0.01	0.01	0.01	**0.61**	**0.47**

Data are median gH1-specific cytokine responses, pg/ml, measured by 15-plex assay in supernatants from PBMC cultures that were stimulated with peptide libraries specific for gH1 for 8 days. Boldface and underlined text represents values for which there is statistical evidence of higher median values compared with median values at baseline. IL-2 in the adjuvanted group possessed statistically lower median values compared with median values at baseline. In the non-adjuvanted group between one and two doses of vaccine statistically lower median values were observed for MIP-3α.

Exposure to the Qbeta-peptides resulted in increases in IFN-γ, IL-17A, IL-17F and IL-5 by PBMCs from individuals receiving adjuvanted or non-adjuvanted Qbeta vaccines. The adjuvanted vaccine also induced Qbeta-specific increases in IL-13, IL-9 and IL-21 (Supplementary Table S5).

### Vaccination with gH1-Qbeta drives an influenza-specific IFN-γ response in a subset of individuals

**Figure 4:**
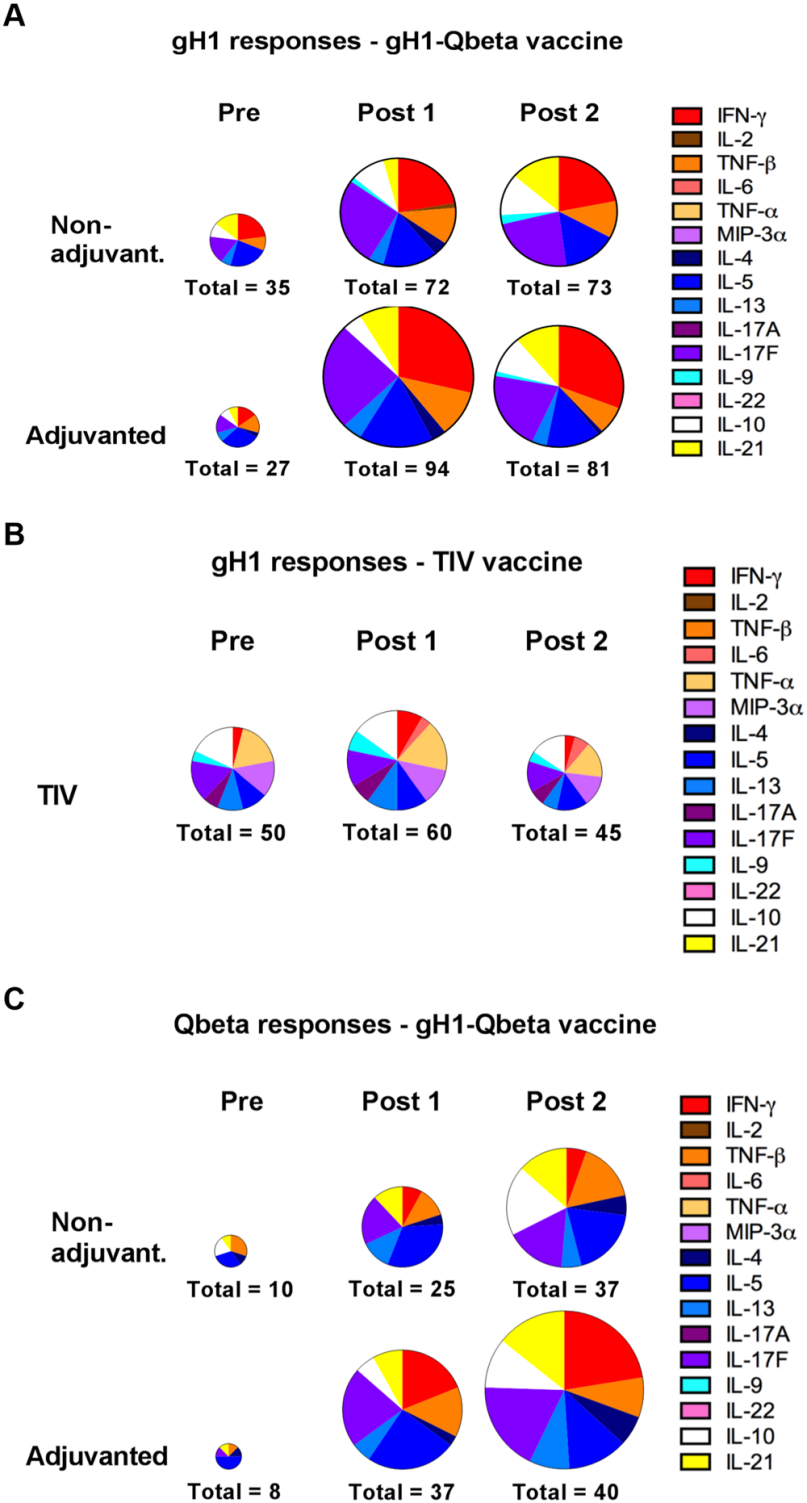
Vaccination with gH1-Qbeta elicits a poly-functional influenza-specific cytokine response. Distribution of positive cytokine responses from subjects vaccinated with (**A**,**C**) non-adjuvanted gH1-Qbeta (n = 37), (**A**,**C**) adjuvanted gH1-Qbeta (n = 43) and (**B**) trivalent influenza vaccine (n = 10). PBMCs were cultured with (**A**,**B**) peptide pools covering the gH1 antigen sequence, and (**C**) peptide pools covering the Qbeta sequence. Levels of the indicated cytokines were measured in the cell culture supernatants after eight days incubation. The size of the charts is proportional to the magnitude of the response (number of positive responses in all subjects for that visit is indicated for each chart; maximum of 150 for TIV, 555 for non-adjuvanted gH1-Qbeta, and 645 for adjuvanted gH1-Qbeta). Labels “Pre”, “Post1” and “Post2”, refer to Day 0, Day 21 and Day 42 post primary vaccination respectively.

Similar to our study in the TIV cohort, in order to look at the quality of response, we normalized the cytokine data in an attempt to look at their relative distributions. Again, cytokine responses were equally distributed, following stimulation with PMA and ionomycin (Supplementary Fig. S9). Also, the distribution of individual positive cytokine responses relative to other cytokines did not fundamentally change following gH1-Qbeta vaccination (Fig. 4A,B).

The large increase in IFN-γ levels observed with the adjuvanted gH1-Qbeta vaccine is also reflected in the number of individual positive IFN-γ responses compared to other cytokines with a trend to more individual positive IFN-γ responses for both gH1- and Qbeta-specific responses (Fig. 4A,C).

**Figure 5:**
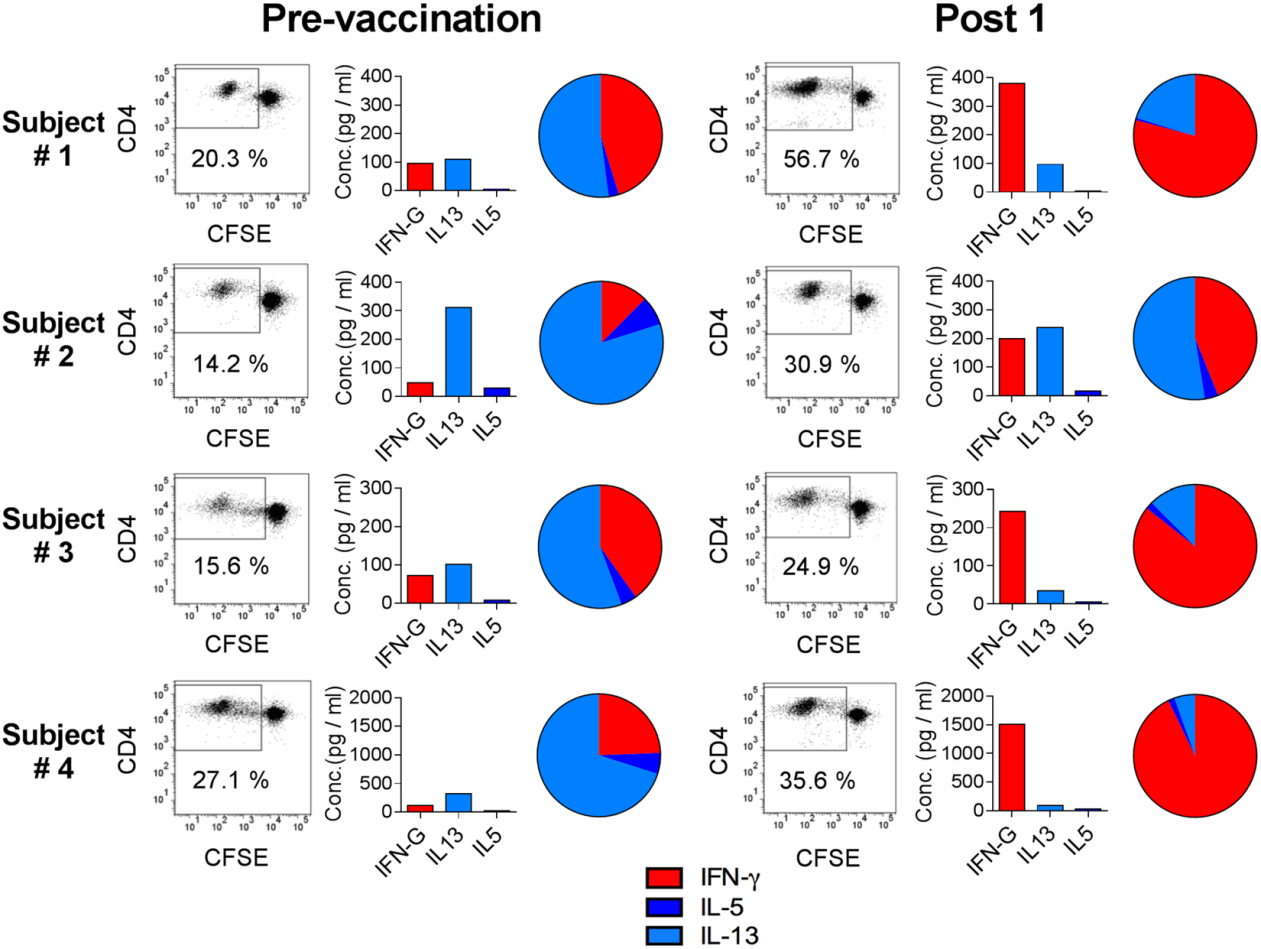
Vaccination with gH1-Qbeta polarizes the quality of the gH1-specific T-cell response in some individuals. Shown are CD4^+^ proliferation and cytokine responses from cultures of PBMCs stimulated with a peptide pool covering the gH1 antigen sequence. PBMCs were collected both before (Pre) and 21 days after administration of the vaccine (Post 1). Data is from individual subjects who received either non-adjuvanted (Subject #2) or adjuvanted gH1-Qbeta (Subject #1, #3 and #4). FACS histogram plots are representative of one of three replicates and percentages of proliferating CD4+ T-cells in the gate region are indicated. Labels “Pre”, “Post1” and “Post2”, refer to Day 0, Day 21 and Day 42 post primary vaccination respectively.

A comparison of T helper type 2 (Th2) cytokines (IL-5 and IL-13) to the T helper type 1 (Th1) cytokine IFN- γ reveal a clear skewing following vaccination with the ratio of IFN-γ to IL-13 and IL-5 increasing following vaccination (Supplementary Fig. S10). This capacity of the gH1-Qbeta vaccine to preferentially elicit IFN-γ is seen in a subset of individuals whose pre-existing influenza-specific cytokine responses were characterized by the T helper type 2 (Th2) cytokines IL-5 and IL-13 (Fig. 5).

### DDX17, a putative RNA helicase, is down-regulated following vaccination

**Figure 6:**
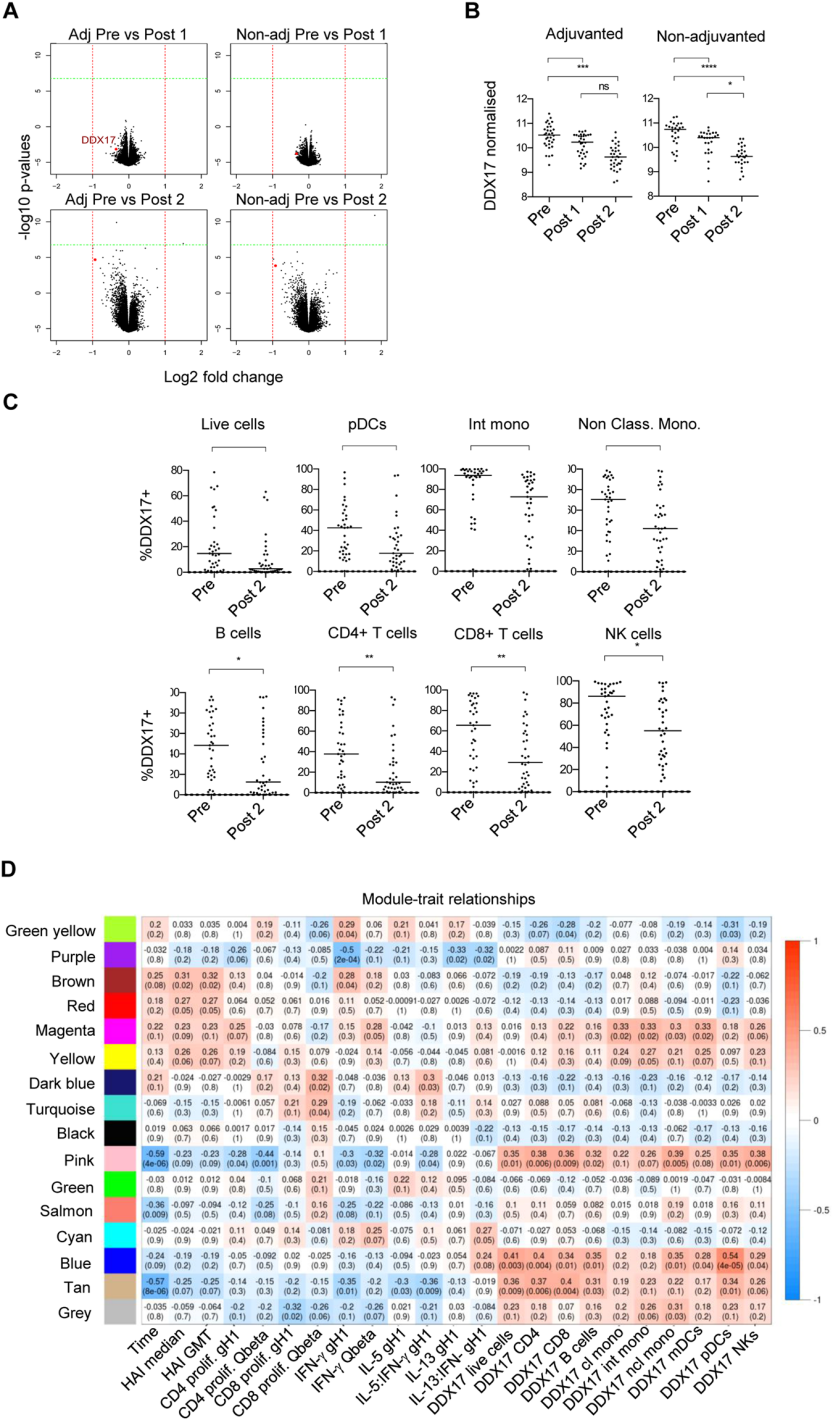
DDX17 gene expression is downregulated post vaccination with gH1-Qbeta and WGCNA/BTM analysis reveals clusters of genes associated with downregulation of DDX17 and upregulation of HAI and CD4 T-cell proliferative responses. (**A**) Volcano plot comparing mRNA microarray data in whole blood samples from subjects at the pre-vaccination timepoint and at 21 days after administration of vaccine [top plots; Pre vs Post 1] and comparing pre-vaccination to 42 days after administration of the vaccine [bottom plots; Pre vs Post 2]. Subjects vaccinated with adjuvanted (left column plots) and non-adjuvanted (right column plots) gH1-Qbeta are depicted as labelled. (**B**) Normalized DDX17 mRNA levels from microarray analysis of whole blood from subjects vaccinated with adjuvanted or non-adjuvanted gH1-Qbeta at pre-vaccination and 21 days following the first (Post 1) and second (Post 2) vaccination. Horizontal bars represent the median value. Friedman test. ****P < 0.0001; ***P < 0.001; *P < 0.05 by Dunn’s test. ns, not significant. (**C**) PBMCs from the cohort vaccinated with non-adjuvanted gH1-Qbeta were analysed by flow cytometry for DDX17 protein expression in a number of identified immune cell populations. Horizontal bars represent the median value. Wilcoxon test. **P < 0.01; *P < 0.05; ns, not significant. (**D**) WGCNA analysis clustered genes with similar expression patterns into modules with randomly assigned colors. Each module was tested for correlation to traits of interest. Pearson correlation coefficients are stated in each box, with p values in brackets. Labels “Pre”, “Post1” and “Post2”, refer to Day 0, Day 21 and Day 42 post primary vaccination respectively.

We next examined the whole blood transcriptome of individuals receiving the gH1-Qbeta vaccination. We analyzed the adjuvanted and non-adjuvanted groups and therefore identified two lists of differentially expressed genes (DEGs) 42 days post vaccination, relative to expression at day 0 (Fig. 6A). When DEGs were filtered based on a fold change threshold of 2, DDX17, a putative RNA helicase, was identified as being significantly down regulated following vaccination in adjuvanted and non-adjuvanted groups (Fig. 6A,B). Flow cytometry analysis on PBMCs revealed that DDX17 protein expression was reduced on a number of leukocyte subsets including pDCs, intermediate and non-classical monocytes, B-cells, CD4^+^ T-cells, CD8^+^ T-cells and NK cells (Fig. 6C). DDX17 expression was also reduced in T-cell subpopulations including central and effector memory CD4+ T-cells following vaccination (Supplementary Fig. S11).

### A transcriptional signature to vaccination correlates with DDX17 expression HAI titer and the influenza-specific IFN-γ T-cell response

We then studied the regulation of groups of genes via a weighted correlation network analysis (WGCNA) to identify clusters of genes whose change in expression was associated with each other. These clusters of genes were assessed for correlations to HAI titer, T-cell responses and DDX17 expression and compared to previously identified Blood Transcriptional Modules (BTMs)^23^ to identify biological functions associated with the gene clusters (Fig. 6D). Multiple gene clusters were positively correlated with HAI titer, including a module (indicated in brown) enriched for genes related to IL-3 signaling, protein export, vitamin B biosynthesis and CoA synthesis. Notably, a specific cluster, indicated in pink, which is enriched in CORO1A-DEF6 network genes, demonstrated reduced activity following vaccination and correlated positively with DDX17 expression, and negatively with HAI titer and influenza-specific IFN-γ cytokine response.

## Discussion

The 2009 H1N1 pandemic highlighted the global threat posed by influenza A viruses and has sparked a flurry of interest in the development of vaccines to prepare for future, and potentially more serious pandemics^24–30^. Effective influenza vaccination is currently assessed by anti-influenza antibody levels due to the accepted and established importance of humoral immunity on protection^4^. Studies have shown that cellular responses play an important role in protection, with associations drawn between pre-existing IFN-γ -producing influenza-specific CD4^+^ and CD8^+^ T-cells and less severe disease^2,3^. Since the current TIV vaccine is inefficient at driving T cell activation^6–12,14–16,18^, a need exists for strategies which can engage both humoral and cell mediated immunity. Herein, we present a novel VLP vaccine able to induce seroconversion and elicit influenza-specific T-cell responses.

In pre-clinical studies we have demonstrated that novel *E*. *coli* derived VLP vaccines displaying the influenza HA globular domain (gH1) induced IFN-γ -producing gH1-specific T-cells characteristic of a potent Th1 response^30^. This response was found to be dependent on ssRNA, a TLR7 agonist, contained within the VLP and the Th1 polarized response persisted in the presence of Th2 polarizing adjuvants such as alum. In addition this preclinical work revealed that the vaccine was able to induce humoral responses comparable to commercial egg-produced vaccines. A Phase I clinical trial showed that humoral responses could also be elicited in humans with the vaccine achieving the primary endpoint of 60% seroconversion^22^. In the current work we demonstrate that, in addition to the induction of antibody responses, the *E*. *coli* derived VLP vaccine gH1-Qbeta elicits gH1-specific T-cell proliferation and production of the T helper 1 cytokine IFN-γ. These responses were not observed in a cohort of individuals vaccinated with the TIV vaccine, although it should be noted that this TIV cohort is smaller and studied separately as part of an independent clinical study. Whether the T-cell responses elicited by our VLP vaccine are equivalent to the protective pre-existing responses reported in the studies of Wilkinson *et al*.^2^ and Sridhar *et al*.^3^ remains unknown. To date a correlation between vaccine-induced T-cell responses and protection against influenza infection has not been demonstrated. To answer this question, further clinical studies are needed.

The T-cell responses described in the studies above are focused on the measurement of a single effector function, namely IFN-γ production by T-cells. By expanding the number of cytokines measured we have aimed to reveal a larger fraction of the antigen-specific response. Measuring a greater proportion of the types of cytokine responses gives information on how the quality of responses changes following vaccination. Notably it reveals the difficulty in changing pre-existing responses to an antigen with vaccination. In addition, it provides information not just about desirable responses, in this case the production of the Th1 cytokine IFN-γ, but also about undesirable responses. An example of such a response is the production of IL-10, which has been associated with reduced protection against influenza infection^31^. Notably we observed that both the TIV and gH1-Qb vaccines resulted in increased production of IL-10 when PBMCs from vaccinated individuals were cultured *ex vivo*. The ability of the gH1-Qbeta VLP vaccine to influence the quality of the cytokine response was further emphasized in specific individuals whose pre-existing influenza responses were characterized by a higher level of the Th2 cytokines IL-5 and IL-13 over the Th1 cytokine IFN-γ. Following vaccination, in addition to an increasing the magnitude of the response, the quality of the cytokine response in these individuals trended towards a more Th1 biased response characterized by the production of IFN-γ. An important caveat that should be highlighted when drawing conclusions from the studies presented here, is that although the cytokines measured are characteristic of polarized T cells, the possibility exists that since the PBMCs were cultivated for eight days, the cytokines initially produced by activated T cells could also induce cytokine production by other lymphocyte subsets. To confirm the cellular source of the cytokines, further clinical studies are needed using alternative techniques such as intracellular cytokine flow cytometry. Blood transcript gene analysis from vaccinated subjects identified DDX17 as a significantly down regulated gene following vaccination with gH1-Qbeta. DDX17 is an RNA helicase involved in transcription and splicing. It is required by H1N1 polymerase for viral replication, therefore making this molecule an interesting candidate for further investigation either as a biomarker or drug target^32^. WGCNA analysis identified multiple gene clusters that correlated with HAI titer. One specific cluster was shown, by BTM analysis^33^, to be enriched in genes from the CORO1A-DEF6 network and correlated with HAI titer as well as the IFN-γ T-cell response and DDX17 expression. Coronin1A is the predominant regulator of the actin cytoskeleton in lymphocytes and its deficiency in humans is associated with susceptibility to infection^34^. DEF6 is a regulatory protein that modulates interferon regulatory factor 4 (IRF4) and has been shown in mice to control the ability of IRF 4 to regulate IL-21 B-cell responsiveness^35^. Further studies are required to explore the involvement of this network in the vaccine response.

Overall, this study showed that both non-adjuvanted and adjuvanted formulations of the gH1-Qbeta vaccine induced influenza-specific CD4^+^ and CD8^+^ T-cell responses and influenza-specific induction of a number of cytokines including anti-viral IFN-γ. Transcriptome analysis revealed gene signatures that correlate with the vaccine response. Further studies including a planned human challenge study with the gH1-Qbeta VLP vaccine will reveal any protective capability against influenza infection as well as the role of T-cell responses in protection and allow validation of the gene signatures identified here.

## Methods

### Study Design

A cohort of ten healthy volunteers aged between 21 and 56 years of age received the Trivalent Influenza Vaccine (TIV) (Approval #CIRB2010/720/E). The vaccine contained 15 µg HA from an A/California/7/2009 (H1N1)pdm09-like virus, an A/Perth/16/2009 (H3N2)-like virus and a B/Brisbane/60/2008-like virus. All volunteers received two doses of the vaccine, intramuscularly, 21 days apart.

In a separate study (Approval #CIRB2012/906/E) 84 healthy volunteers aged between 21 and 64 years of age received two doses of gH1-Qbeta virus-like particle pandemic influenza A (H1N1) 2009 vaccine (HSA CTC1300092). More details of this clinical study are reported in Low *et al*. 2009^22^. Briefly this was a double-blinded, 1:1 randomized Phase 1 healthy volunteer study conducted at two sites in Singapore. All participants received two intramuscular injections of 100 µg vaccine (42 µg HA) per dose, 21 days apart, either non-adjuvanted or adjuvanted with 2% alhydrogel, in a total volume of 500 µl per injection.

In the TIV study, subjects were excluded if they had received any previous vaccine that contained influenza A/California 2009 while in the gH1-Qbeta study any receipt of a seasonal influenza vaccination was an exclusion criteria. In addition, subjects with HAI titers >1:40 at screening were excluded.

Study approval was obtained from the Singapore Health Sciences Authority (HSA) and the Centralized Institutional Review Board (CIRB Ref: 2010/720/E and 2012/906/E) and the study was performed in agreement with the International Conference on Harmonisation guidelines on Good Clinical Practices, laws and regulatory requirements in Singapore. A written informed consent was obtained from each subject prior to screening.

### HAI and Microneutralization (MN) assays

Serum samples from the TIV cohort were analyzed using standard, non-validated HAI and MN assays. For the gH1-Qbeta study validated HAI assays were performed by Southern Research Institute (Birmingham, AL).

### Antigen specific T-cell proliferation assay

Frozen/thawed PBMC were labelled with 1 μM CFSE and co-cultured in complete culture medium with either overlapping peptide pools (15-mers, with a 4-aa lag, 1 μg/ml per peptide) or the vaccine Fluarix (1 μg/ml) (10^5^ cells per well). At day 8, cells were stained with anti-CD3AlexaFluor700^®^ (UCHT1; Becton Dickinson), anti-CD8APC-Cy7 (SK1; Becton Dickinson), anti-CD4PE-Cy7 (RPA-T4; BioLegend), anti-CD45Pacific Orange (HI30; Invitrogen), anti-CD19APC (HIB19; BioLegend) and 7AAD viability staining solution (eBiosciences), and analysed by flow cytometry using a LSR Fortessa (BD). For the TIV cohort, cells were also stained with anti-PD1PE (EH12.2H7; BiolLegend), anti-ICOSPE-Cy7 (C398.4A; BioLegend) and anti-CXCR5A647 (RF8B2; Becton Dickinson). Cytokine measurements with bead-based cytokine multiplex analysis (Luminex) were performed at day 8. More detailed method in supplementary material.

### Flow cytometry analysis of PBMCs

PBMCs were incubated with LIVE/DEAD® Fixable Aqua stain (ThermoFisher Scientific) followed by anti-BDCA-1 APC/Cy7 (L161; BioLegend). anti-BDCA2 PE (AC144; Miltenyi Biotec), anti-CD3 BUV395 (UCHT1; Becton Dickinson), anti-CD4 BUV737 (SK3; Becton Dickinson), anti-CD8 PE/Cy7 (53–6.7; Becton Dickinson), anti-CD11c BV421 (B-ly6; Becton Dickinson), anti-CD14 BV711 (MΦP9; Becton Dickinson), anti-CD16 PE/Cy5 (3G8; BioLegend), anti-CD19 BV786 (SJ25C1; Becton Dickinson), anti-CD56 PE/CF594 (B159; Becton Dickinson) and anti-HLA-DR APC (L243; Becton Dickinson). Cells were fixed and permeabilised using Foxp3/Transcription Factor Staining Buffer set (eBioscience) and DDX17 detected in a two step process using rabbit anti-DDX17 primary (EPR13807[B]; Abcam) and by polyclonal secondary donkey anti-rabbit DyLight®488 (Abcam). Cells were analysed by flow cytometry using a LSRII analyser (BD Biosciences). Representative gating for DDX17 can be seen in Supplementary Fig. S13.

For T-cell differentiation, PBMCs were stained with LIVE/DEAD® Fixable Near-IR stain (ThermoFisher Scientific) followed by anti-CD3 BUV395 (UCHT1; Becton Dickinson), anti-CD25 BUV737 (2A3;Becton Dickinson), anti-CD8 BV650 (RPA-T8; Becton Dickinson), anti-CD4 PE (RPA-T4; Becton Dickinson), anti-CD45 RA ECD (2H4LDH11LDB9; Beckman Coulter) and anti-CCR7 PE-Cy7 (3D12; Becton Dickinson). Intracellular staining was performed for anti-FoxP3 ef450 (PCH101; eBioscience) and anti-DDX17 (as described above).

### Microarray analysis

RNA was extracted from whole blood collected in PAXgene^®^ Blood RNA tubes (BD Biosciences) using the Rneasy Mini Kit (Qiagen). RNA was amplified using TotalPrep-96 RNA Amplification Kit (Applied Biosystems) and hybridised to an Illumina Sentrix BeadChip Array HumanHT-12_v4_BeadChip. The BeadChip was scanned on a BeadArray Reader (Illumina) and analysed using BeadScan Software V2011.1 (Illumina). Expression data was normalized and processed with the Bioconductor lumi R package. Weighted correlation network analysis was carried out in the WGCNA R package with 4223 genes, after genes with low mean expression and low standard deviation of expression were filtered. BTM module enrichment tests, using modules previously described^33^ were conducted in R using Fisher’s exact tests with Bonferroni adjustment.

## Electronic supplementary material

Supplementary Materials, Figures and Tables

## Data Availability

All the data that generated, analysed and used to support the findings of this study are available from the corresponding author upon request.
