# Supplementary material for: Induction of Human T-cell and Cytokine Responses Following Vaccination with a Novel Influenza Vaccine
Source: Sci Rep. 2018 Dec 20;8:18007. doi: 10.1038/s41598-018-36703-7 (PMC6301966; doi:10.1038/s41598-018-36703-7)
Supplement: Supplementary file 1 — Supplementary Materials, Figures and Tables [file 41598_2018_36703_MOESM1_ESM.docx]

**Induction of Human T-cell and Cytokine Responses Following Vaccination with a Novel Influenza Vaccine**

**Authors:**  David A. G. Skibinski^1,2^, Leigh Ann Jones^1^, Yuan O. Zhu^3^, Lin Wu Xue^1^, Bijin Au^1^, Bernett Lee^4^, Ahmad Nazri Mohamed Naim^3^, Audrey Lee^1^, Nivashini Kaliaperumal^1^, Jenny G. H. Low^5,6^, Lawrence S. Lee^7,8^, Michael Poidinger^4^, Philippe Saudan^9^, Martin Bachmann^10^, Eng Eong Ooi^11^, Brendon J. Hanson^12^, Veronica Novotny-Diermayr^13^, Alex Matter^13^, Anna-Marie Fairhurst^4^, Martin L. Hibberd^3^ and John E. Connolly^1,2*^

Supplementary Materials:

Supplementary Methods:

*Antigen specific T-cell proliferation assay*

PBMCs were isolated using BD Cell Preparation Tube with EDTA (Becton Dickinson), and were frozen at a controlled rate in fetal bovine serum (FBS) containing 10% DMSO. Cryopreserved PBMCs were thawed at 37°C and washed in RPMI 1640 medium (Gibco) containing 10% human AB serum (Life Technologies), 15 mM HEPES (Gibco), 1% Non-essential amino acid (Gibco), 1 mM Sodium Pyruvate (Gibco), 1% Penicillin/Streptomycin (Gibco), 2 mM L-glutamine (Gibco) and 50 mM ß2-mercaptoethanol (Sigma).

Once thawed, PBMCs were labelled with CFSE (5-(and-6)-carboxyfluorescein diacetate, succinimidyl ester) and cocultured in deep-well plates (Nunc; 10^5^ cells per well) with overlapping peptide pools (15-mers, with a 4 amino acid lag; 1 µg/ml per peptide). Peptide pools covering the influenza A/California/7/2009 HA, MP1 and NP domains were purchased from JPT (Supplementary Tables S6, S7 and S8), and custom peptides specific for the gH1 domain were purchased from 1st Base (Supplementary Tables S9). Custom peptides specific for the Qbeta domain, were purchased from Genscript, and used to probe responses to the VLP region of the gH1-Qbeta vaccine (Supplementary Tables S10). Peptides were diluted in 50% acetonitrile to a concentration of 10 mM and stored at -80°C until use. An equal amount of 50% Acetonitrile was added to control cultures. The vaccine Fluarix (1 µg/ml) was used to monitor vaccine-specific responses for the cohort that received the TIV vaccine to monitor vaccine-specific responses. Stimulation of PBMCs with PMA (50 ng/ml; Sigma, B139) /ionomycin (1 mg/ml; Sigma Cat#I0634) was used as positive control and induced frequencies proliferation for CD4^+^ and CD8^+^ T-cells above 15 % and 25 % in all subjects respectively. Peptide/ culture plates were incubated in a humidified incubator at 37 °C and 5 % CO2.

On day 8, culture supernatant was retrieved for measurement of cytokine concentrations and cells stained for FACS analysis. For FACS, cells were incubated with anti-Fc receptor blocking antibody (eBiosciences) and subsequently with anti-CD3AlexaFluor700^®^ (UCHT1; Becton Dickinson), anti-CD8APC-Cy7 (SK1; Becton Dickinson), anti-CD4PE-Cy7 (RPA-T4; BioLegend), anti-CD45Pacific Orange (HI30; Invitrogen), anti-CD19APC (HIB19; BioLegend) and 7AAD viability staining solution (eBiosciences). For the TIV cohort, a set offTIV stimulated samples were incubated with anti-Fc receptor blocking antibody (eBiosciences) and subsequently with anti-CD3AlexaFluor700^®^ (UCHT1; Becton Dickinson), anti-CD8APC-Cy7 (SK1; Becton Dickinson), anti-CD4Qdot® 655 (RPA-T4; eBiosiences), anti-CD4PE-Cy7 (RPA-T4; BioLegend), anti-CD45Pacific Orange (HI30; Invitrogen), 7AAD viability staining solution (eBiosciences), anti-PD1PE (EH12.2H7; BiolLegend), anti-ICOSPE-Cy7 (C398.4A; BioLegend) and anti-CXCR5A647 (RF8B2; Becton Dickinson). Cells were analysed by flow cytometry using a LSR Fortessa analyzer (BD Biosciences). The gating strategy can be seen in Fig. S1.

For each sample three replicates were analysed by flow cytometry with the subsequent supernatants pooled for Luminex analysis as a single supernatant. For Luminex, 15 cytokine and chemokine concentrations were measured: interleukin 2 (IL-2), interleukin 4 (IL-4), interleukin 5 (IL-5), interleukin 6 (IL-6), interleukin 9 (IL-9), interleukin 10 (IL-10), interleukin 13 (IL-13), interleukin 17A (IL-17A), interleukin 17F (IL-17F), interleukin 21 (IL-21), interleukin 22 (IL-22), interferon gamma (IFN-γ), tumour necrosis factor α (TNF-α), tumour necrosis factor β (TNF-β) and macrophage inflammatory protein 3 α (MIP-3α) using a human “Milliplex” premixed kit according to the manufacturer’s instructions (no. HTH17MAG-14K-15, Millipore). Cytokines were measured in pooled 25-μL supernatants. Multiplex plates were read on the MAGPIX Luminex reader and results were analysed using Bioplex Manager 6.1 software. The standard curve ranges are as follows: IL-4 and IL-17F are 0.02-100ng/ml; IFNγ is 10-40,000 pg/ml; IL-10 is 1-5,000 pg/ml; IL-13 is 7-30,000 pg/ml; IL-2 and IL-17A are 12-50,000 pg/ml; IL-21 and MIP-3α are 5-20,000 pg/ml; IL-22 and TNF-β are 0.04-150 ng/ml; IL-5 is 6-25,000 pg/ml; IL-9 is 9-35,000 pg/ml; IL-6 and TNF-α are 2.5-10,000 pg/ml.

As samples were not all run on a single day, day-to-day variation was assessed by thawing identical control samples from a non-vaccinated donor on each day the assay was performed. Across the assay, the co-efficient of variance for these control PBMCs was 22 %.

*Statistical analysis*

For HAI and MN antibody responses, differences between visits were determined by one way ANOVA on the log transformed titers with the significance level set at 5 %. All ANOVA tests found to be significant were followed up with Tukey’s honest significant difference test to compare between any two groups. The associations between HAI titer and MN titer, and between MN titer and T-cell proliferation for the TIV vaccine was determined by the Spearman rank correlation test.

For the T-cell proliferation analysis (flow cytometry), each data point is the average of three replicates that was background subtracted using the corresponding unstimulated sample. Negative values after background subtraction were designated zero. To determine differences between visits, averaged readings were evaluated with the Friedman test with the significance level set at 5%. All Friedman tests found to be significant were followed up with Dunn’s test to compare between any 2 groups. To determine whether there was a difference in T-cell proliferation between the two gH1-Qbeta vaccine formulations, Mann-Whitney tests were performed and adjusted for multiple testing using the Bonferroni correction.

For the multiplex cytokine analysis, each data point was background subtracted using the corresponding unstimulated sample. Negative values after background subtraction were designated 0.01. To determine differences between visits, averaged readings were evaluated using one-way repeated-measures ANOVA with the visits considered as a within subject term on the log transformed averaged concentrations and corrected for multiple comparisons using the method of Benjamini and Hochberg. T tests were performed on factors found to be significant at the 5% significance level and corrected for multiple comparisons with Bonferroni correction. To determine whether there was a difference in the cytokine levels between the two gH1-Qbeta vaccine formulations, two-way repeated-measures ANOVA was performed on the log transformed titers with the time point as the within subject term and the vaccine as the between subject term. Multiple testing corrections were performed using the method of Benjamini and Hochberg. Where both factors and their interaction were found to be significant at the 5% significance level, T tests were performed to compare between two groups and corrected for multiple comparisons using Bonferoni’s.

For determining the distribution of positive responses from the multiplex cytokine analysis, data was log transformed (Base 10). Positive responses for each individual are those greater than 2 standard deviations above the mean of the background (mean of negative control no peptide stimulation wells for all subjects and visits in the study). The size of the charts is proportional to the magnitude of the response (number of positive responses in all subjects for that visit).

For gH1-Qbeta vaccine formulations associations between T-cell proliferation, cytokine levels and antibody titers were assessed with Spearman Rank correlation co-efficients and corrected for multiple testing using the method of Benjamini and Hochberg.

All statistical tests were deemed to be significant if the P value was less than 0.05.

**
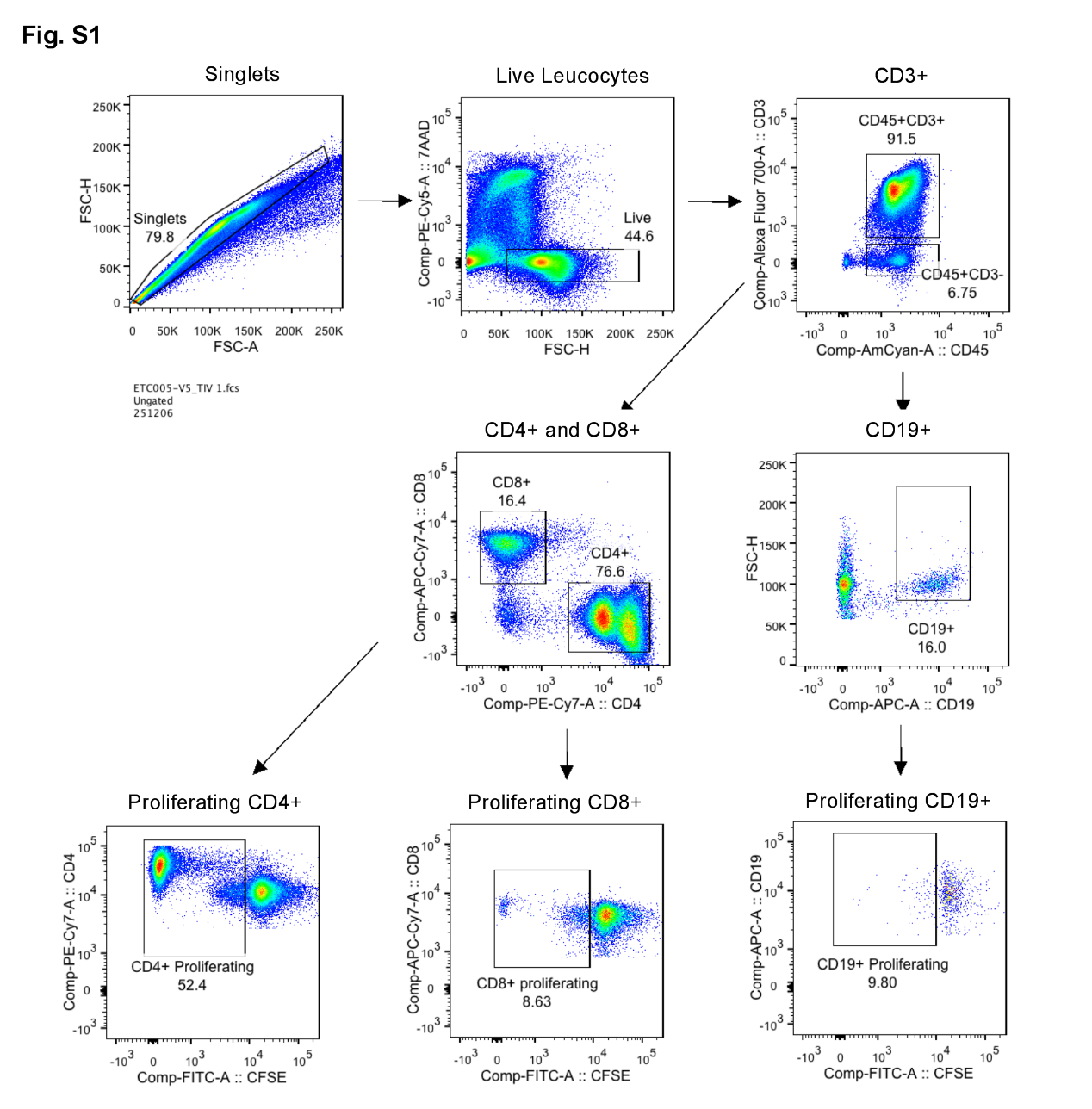
**

**Fig. S1. Representative gating strategy for CSFE-stained PBMCs from a healthy volunteer vaccinated with a single dose of TIV vaccine.** PBMCs were cultured with TIV vaccine for 8 days and then stained for lineage (CD45, CD3, CD4, CD8, CD19) and live/dead markers (7ADD).


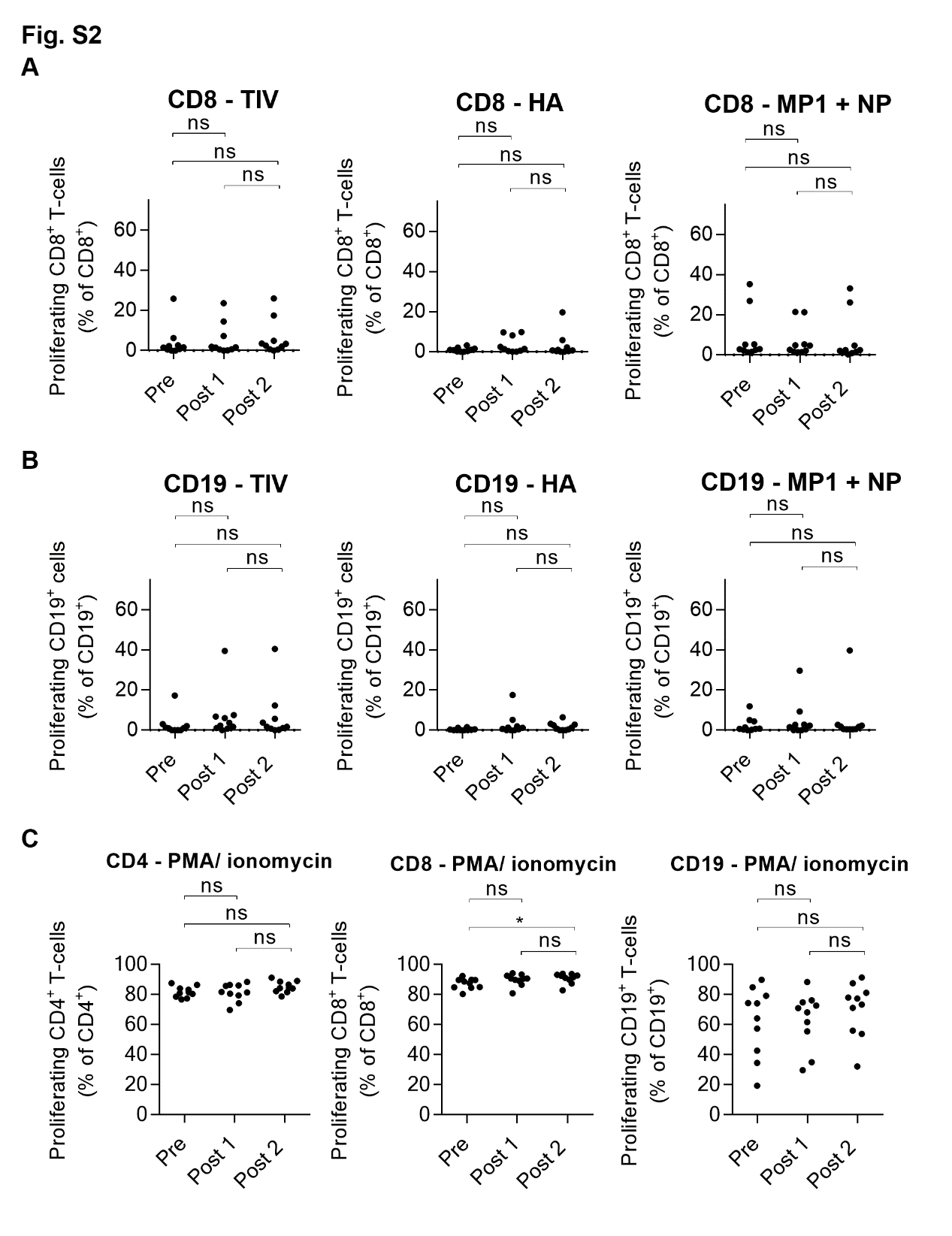


**Fig. S2. Proliferation** of **CD4^+^, CD8^+^ and CD19^+^ cells following trivalent influenza vaccination.** (A, B) Proliferating TIV-, HA-, MP1- and NP- specific CD8^+^ T-cells (A) and CD19^+^ B-cells (B) in cultures of PBMCs isolated from subjects vaccinated with TIV. (C) Proliferation of CD4^+^, CD8^+^ and CD19^+^ cells following PMA and ionomycin stimulation of PBMCs from subjects vaccinated with TIV with PMA and ionomycin. Friedman test. * *P* < 0.05, Dunn’s test. ns, not significant. Labels “Pre”, “Post1” and “Post2”, refer to Day 0, Day 21 and Day 42 post primary vaccination respectively.

**
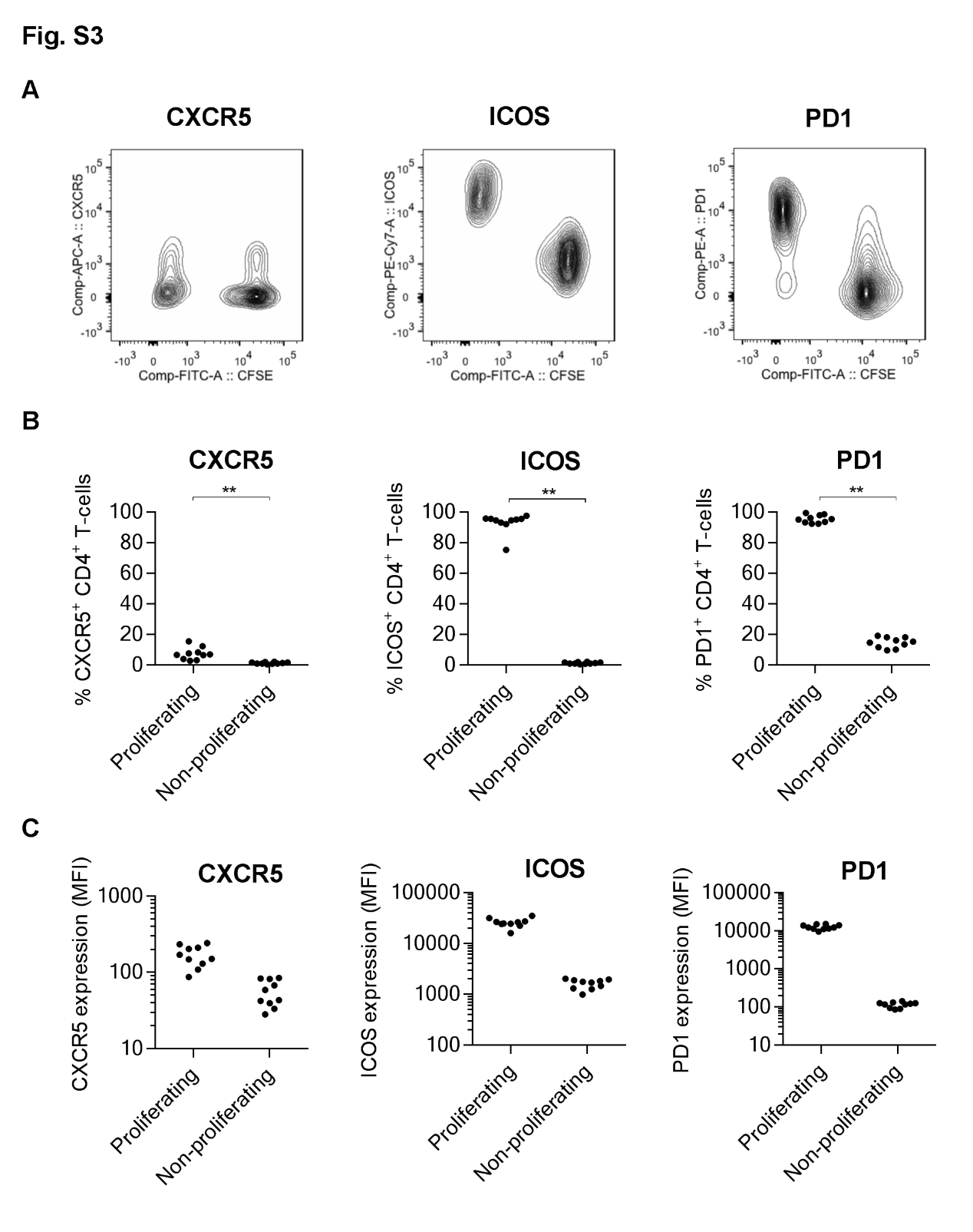
**

**Fig. S3. Expression of CXCR5, ICOS and PD1 on the surface of proliferating (CFSE lo) CD4+ T cells.** (A) Representative histogram plots showing the expression of CXCR5, ICOS and PD1 on the surface of proliferating (CFSE lo) CD4+ T-cells relative to non-proliferating (CFSE hi) CD4^+^ T-cells. PBMCs from a healthy volunteer vaccinated with a single dose of TIV vaccine were stained with CFSE and cultured with TIV vaccine for 8 days. (B) Frequency of proliferating (CFSE lo) and non-proliferating (CFSE hi) CD4^+^ T cells expressing the surface receptors CXCR5, ICOS and PD1. PBMCs, collected 21 days after administration of the first dose of vaccine (Post 1), were stimulated for 8 days with the TIV vaccine. Wilcoxon test. ** *P* < 0.01. (C) Relative expression (MFI) of CXCR5, ICOS and PD1 on the surface of proliferating (CFSE lo) CD4^+^ T-cells relative to non-proliferating (CFSE hi) CD4^+^ T-cells. PBMCs were stimulated for 8 days with TIV vaccine.

**
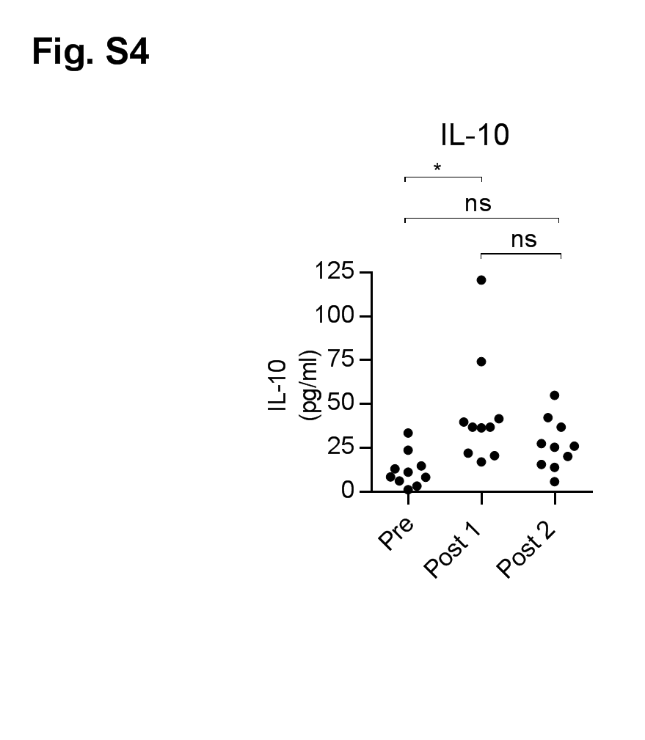
**

**Fig. S4. TIV-specific IL-10 responses following Fluarix vaccination.** One way ANOVA. ** P < 0.01, Benjamini and Hochberg. ns, not significant. Labels “Pre”, “Post1” and “Post2”, refer to Day 0, Day 21 and Day 42 post primary vaccination respectively.

**
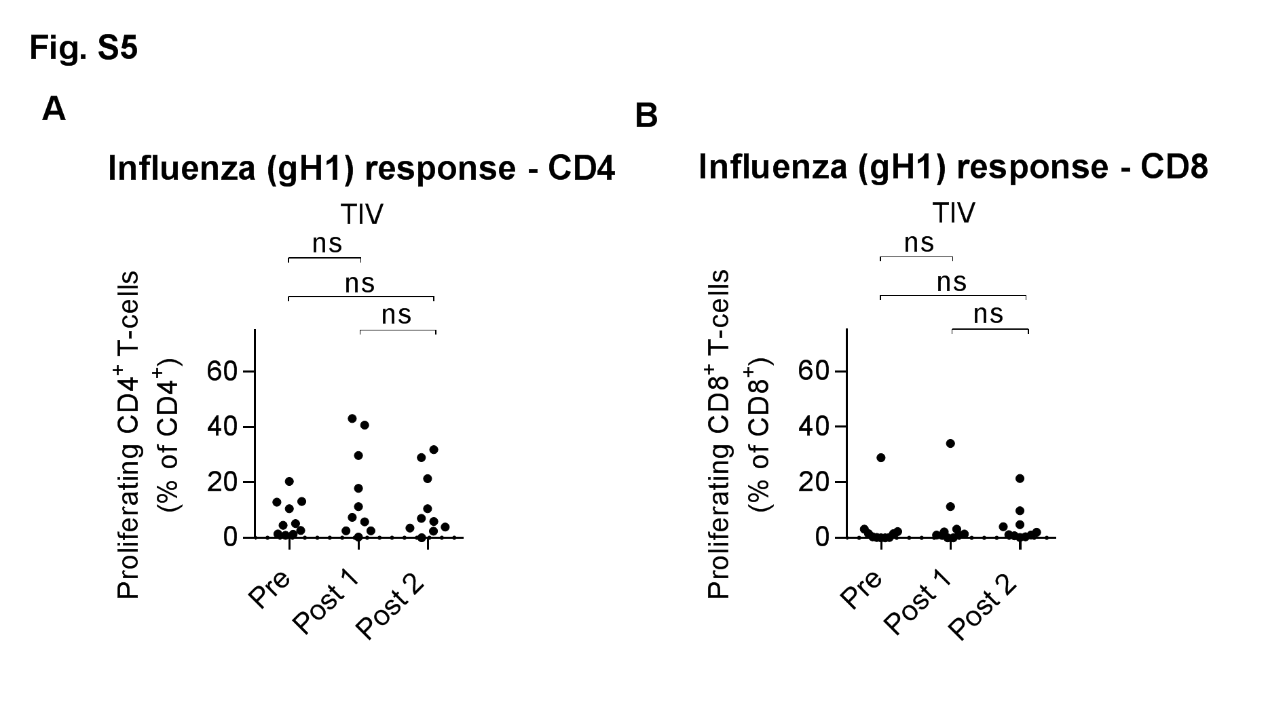
**

**Fig. S5. Proliferating gH1-specific CD4^+^ (A) and CD8^+^ (B) T-cells in cultures of PBMCs isolated from subjects vaccinated with TIV.** ns, not significant. Labels “Pre”, “Post1” and “Post2”, refer to Day 0, Day 21 and Day 42 post primary vaccination respectively.

**
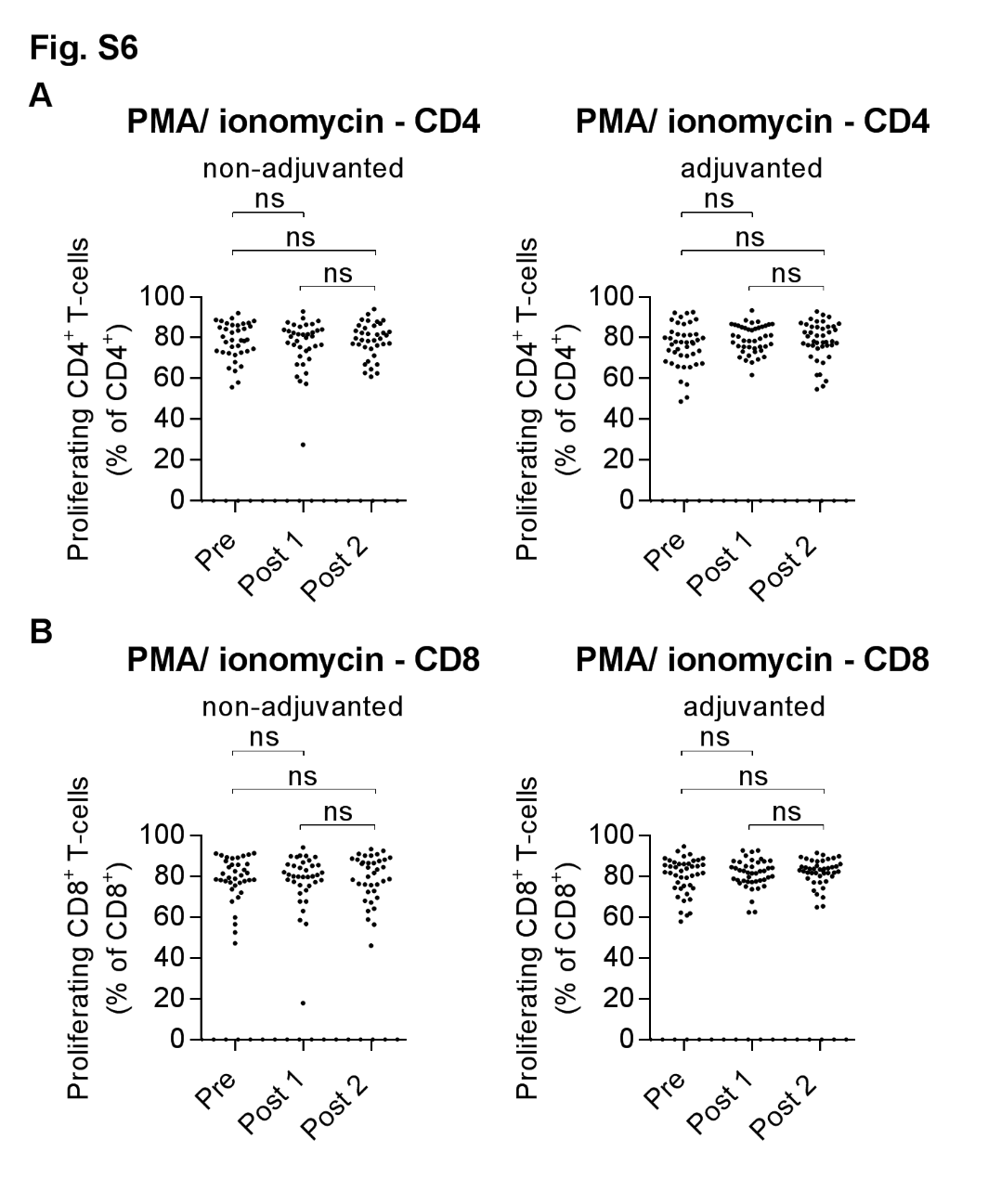
**

**Fig. S6. Proliferation** **CD4^+^ and CD8^+^ T-cells following PMA and ionomycin stimulation of PBMCs from subjects vaccinated with gH1-Qbeta.** Shown are the percentage of CD4^+^ (A) and CD8^+^ (B) T-cells that are proliferating in cultures of PBMCs that were stimulated for 8 days with PMA and ionomycin. ns, not significant. Labels “Pre”, “Post1” and “Post2”, refer to Day 0, Day 21 and Day 42 post primary vaccination respectively.

**
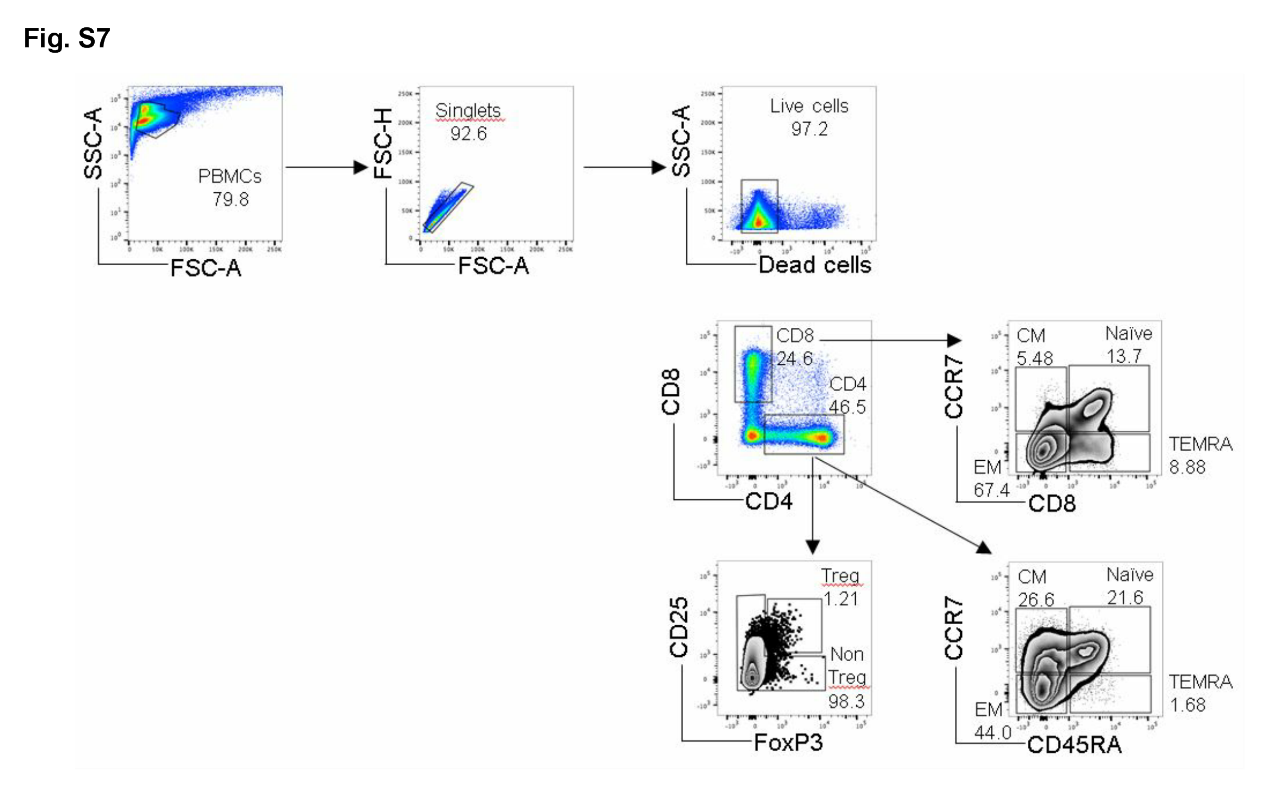
**

**Fig. S7. Representative gating strategy for the identification of T-cell sub-populations.**

**
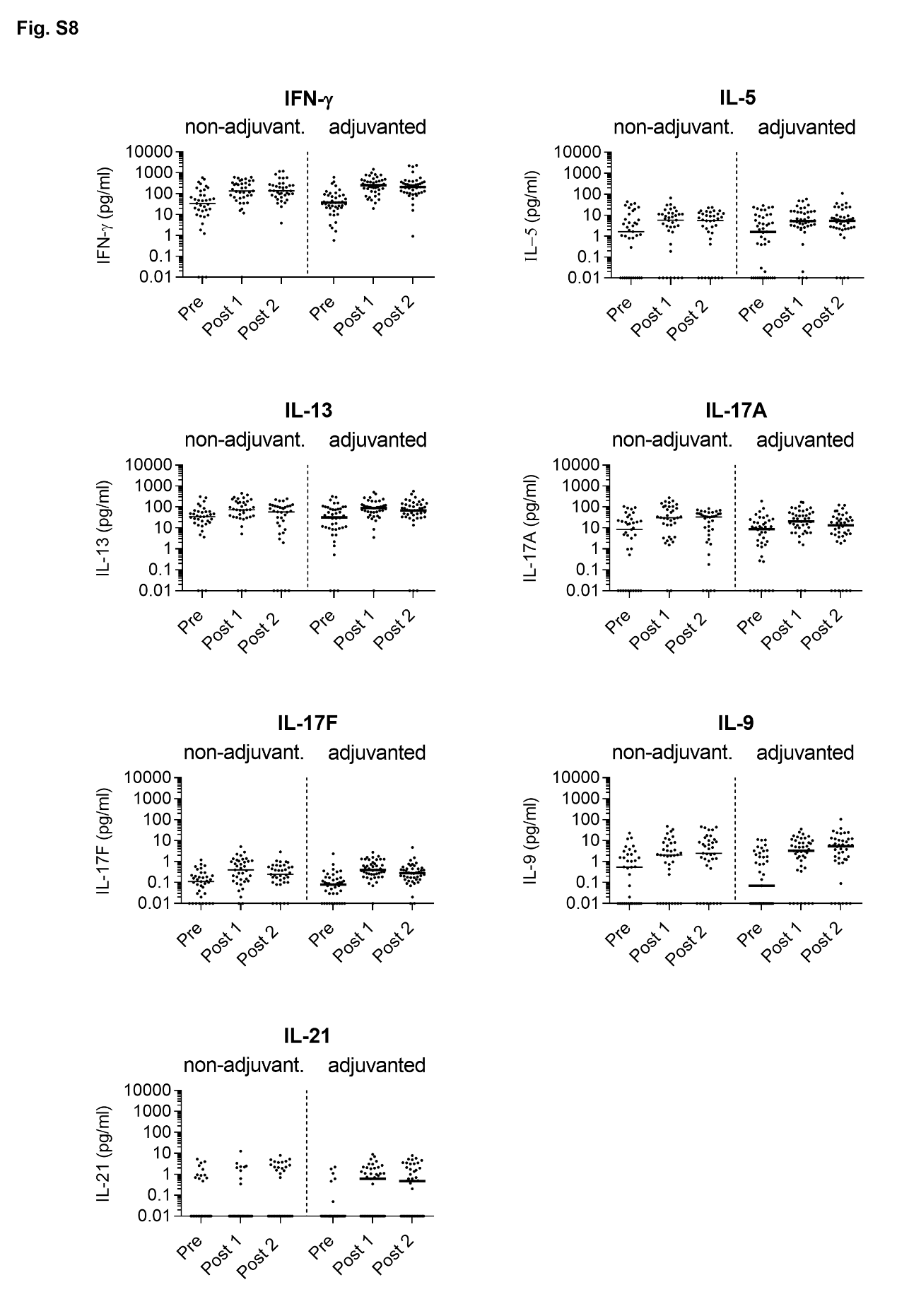
**

**Fig. S8. Individual gH1-specific cytokine responses, pg/ml, measured by 15-plex assay in supernatants from PBMC cultures that were stimulated with peptide libraries specific for gH1 for 8 days.** Line represents median response. Labels “Pre”, “Post1” and “Post2”, refer to Day 0, Day 21 and Day 42 post primary vaccination respectively.

**
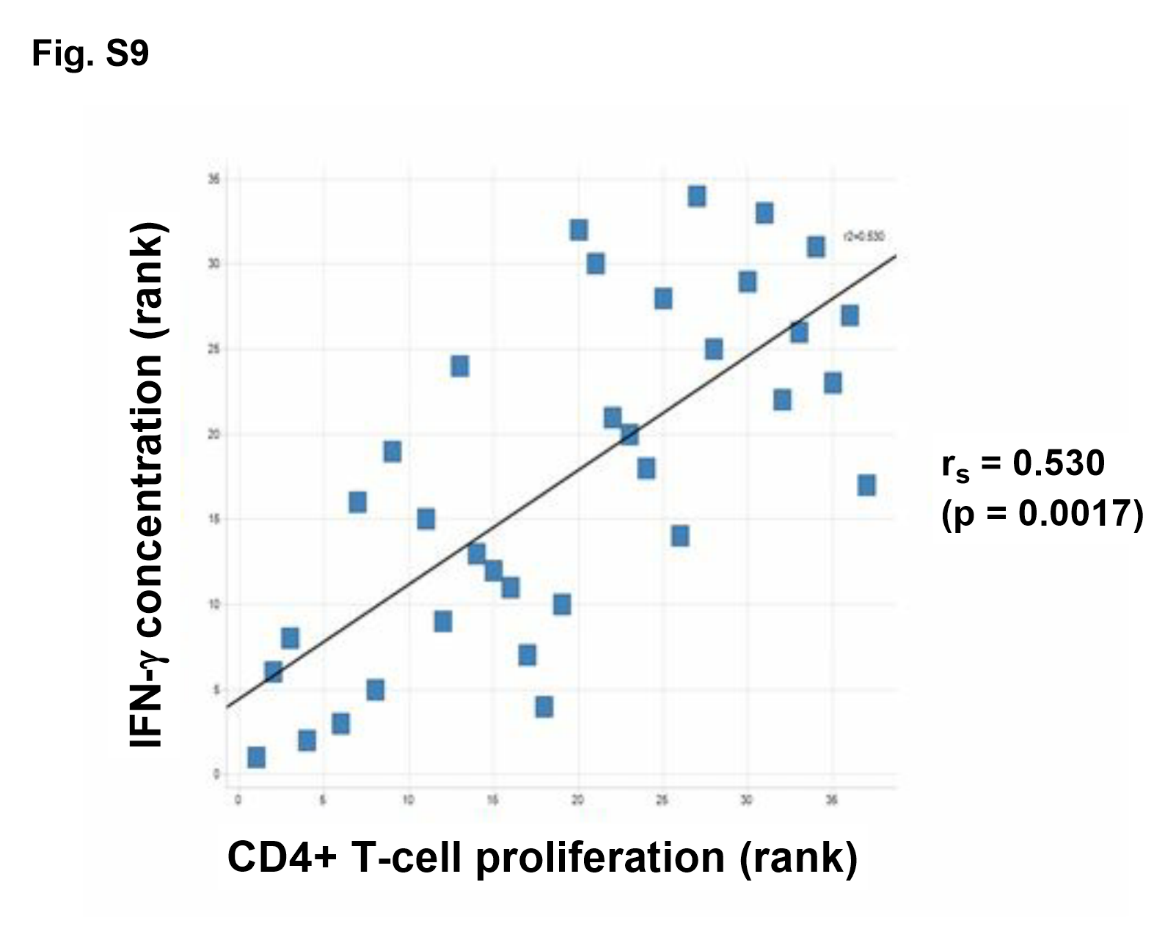
**

**Fig. S9. Correlation between influenza-specific CD4^+^ T-cell proliferation and IFN-γ secretion from PBMCs of subjects vaccinated with a single dose of non-adjuvanted gH1-Qbeta.** PMBCs were cultured with peptides specific for gH1 and both T-cell proliferation and the levels of secreted IFN-γ were measured after eight days incubation. Spearman correlation is shown.

**
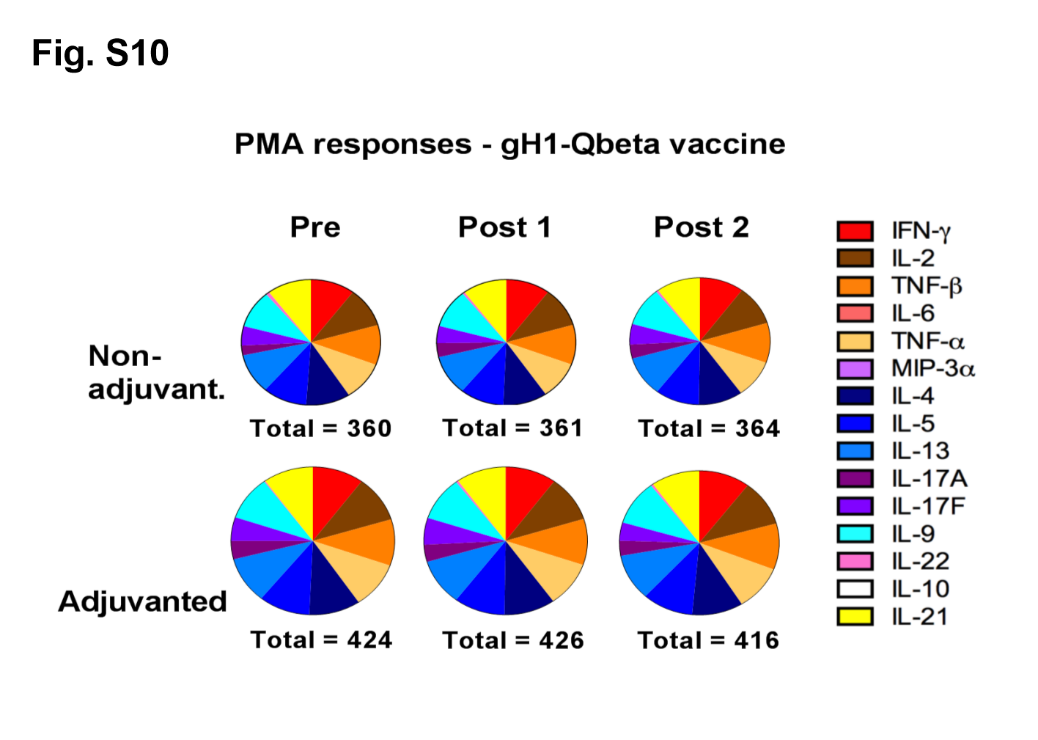
**

**Fig. S10.** **Distribution of positive cytokine responses following PMA and ionomycin stimulation of PBMCs from subjects vaccinated with gH1-Qbeta.** PMBCs were cultured with PMA and ionomycin and levels of the indicated cytokines were measured in the cell culture supernatants after eight days incubation. The size of the charts is proportional to the magnitude of the response (number of positive responses in all subjects for that visit is indicated for each chart; maximum of 555 for non-adjuvanted gH1-Qbeta, and 645 for adjuvanted gH1-Qbeta). Labels “Pre”, “Post1” and “Post2”, refer to Day 0, Day 21 and Day 42 post primary vaccination respectively.

**
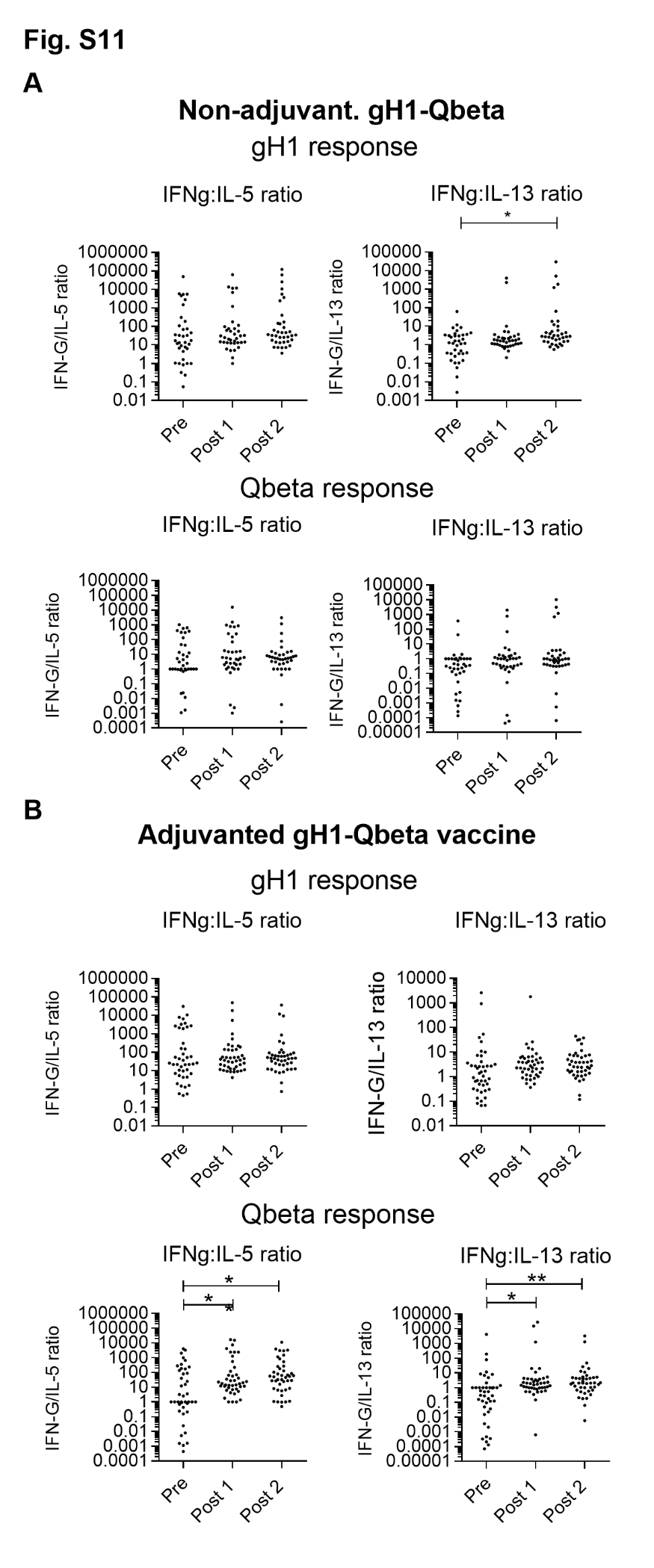
**

**Fig. S11. Ratio of IFN-γ to IL-5, and IFN-γ to IL-13 in cultures of PBMCs isolated from subjects following vaccination with (A) non-adjuvanted, and (B) adjuvanted gH1-Qbeta.** PBMCs were stimulated for 8 days with a peptide pools covering the gH1 and Qbeta antigen sequence. Labels “Pre”, “Post1” and “Post2”, refer to Day 0, Day 21 and Day 42 post primary vaccination respectively.

**
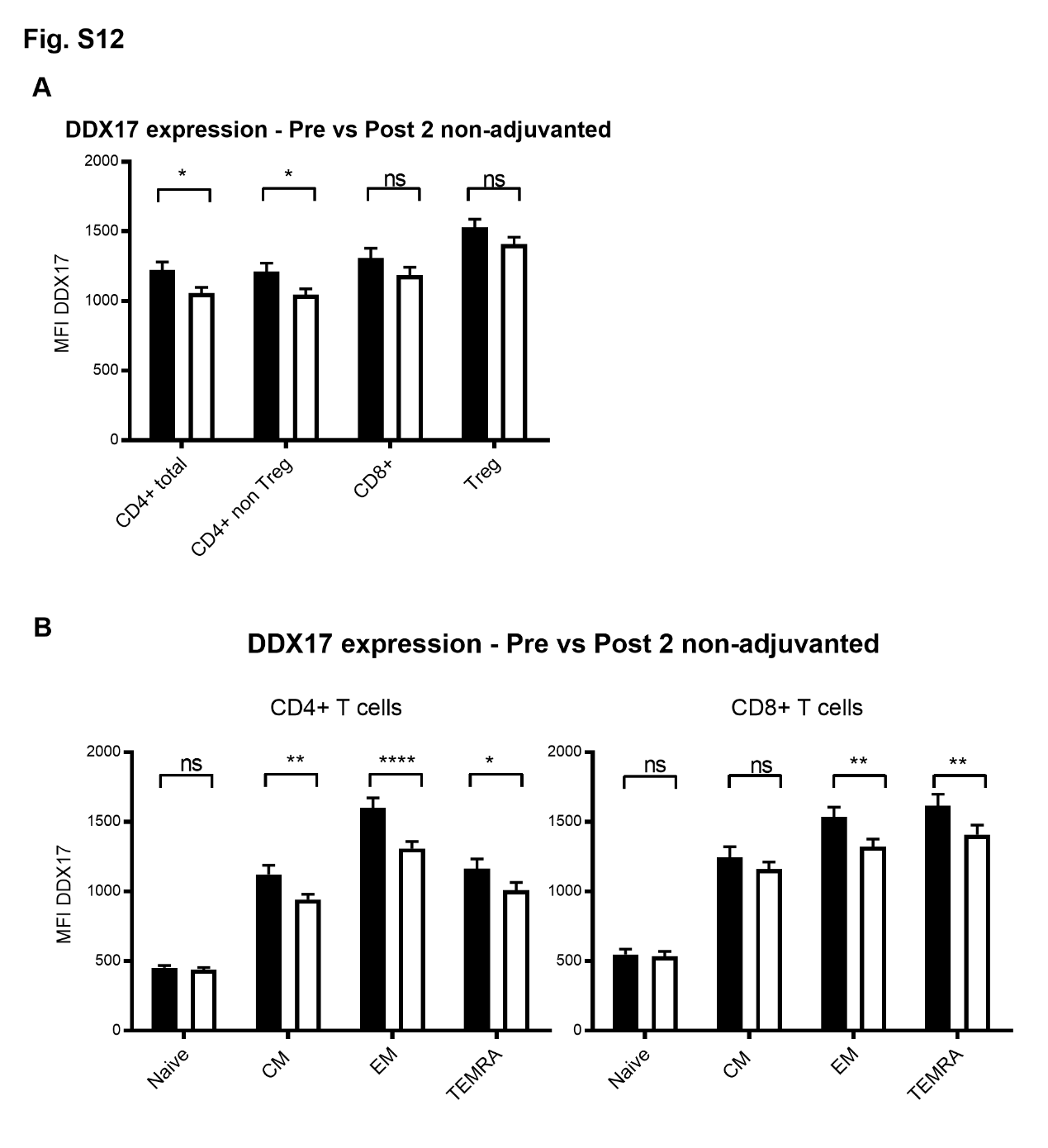
**

**Fig. S12. DDX17 protein expression on T-cell sub-populations.** (A, B) PBMCs from the cohort vaccinated with non-adjuvanted gH1-Qbeta were analysed by flow cytometry for DDX17 protein expression. Repeated measures 2-way ANOVA. ****, P<0.0001; **, P<0.01; *, P<0.05; ns, not significant.

**
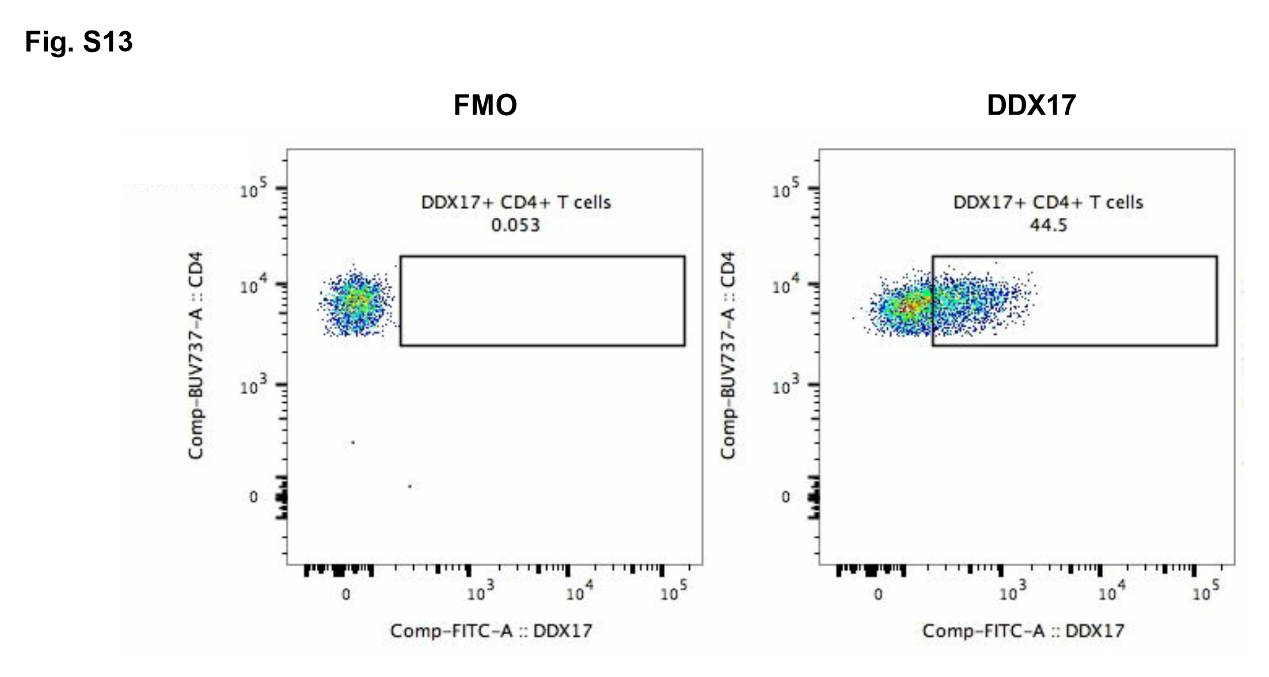
**

**Fig. S13. Representative gating of PBMCs from a healthy volunteer.** PBMCs were gated on CD4^+^ T-cells and gating is shown for the FMO control with polyclonal secondary donkey anti-rabbit alone (left), and for rabbit anti-DDX17 primary together with polyclonal secondary donkey anti-rabbit (right).

**Table S1.** **Influenza antigens identified by mass spectrometry (MALDI-TOF) of the Trivalent Inactivated Influenza Vaccine against A/California/7/2009 (H1N1)pdm09-like virus, A/Perth/16/2009 (H3N2)-like virus and B/Brisbane/60/2008-like virus.** The table reports the accession numbers of the identified proteins, the peptides generated by trypsin digestion and their corresponding Mascot ion scores.

|  | **Peptide sequence** | **Percentage Sequence Coverage** | **Mascot Ion Score** |
| --- | --- | --- | --- |
| **Protein (accession number)** |  |  |  |
| **Matrix protein 1**  **(Q07FX6_9INFA)** | AMEQMAGSSEQAAEAMEVASQAR  GILGFVFTLTVPSER  NTDLEVLMEWLK  QMVTTTNPLIR  SIIPSGPLK  TIGTHPSSSAGLKNDLLENLQAYQK  TRPILSPLTKGILGFVFTLTVPSER | 41.70% | 75.9  91.2  81.9  73.8  33.2  42.3  42.4 |
| **Hemagglutinin**  **(Q4G5Z3_9INFB, Q596G1_9INFB, Q5V9C2_9INFB, Q5V9C3_9INFB, Q5V9C4_9INFB, Q5V9D3_9INFB, Q5V9D4_9INFB, Q5V9D5_9INFB, Q5V9D6_9INFB, Q9WPM4_9INFB)** | ADTISSQIELAVLLSNEGIINSEDEHLLALER  AIGNCPIWVK  CLNCTDLDVALGRPK  DRICTGITSSNSPHVVK  GFFGAIAGFLEGGWEGMIAGWHGYTSHGAHGVAVAADLK  GILLPQKVWCASGR  GSLPLIGEADCLHEK  IVVDYMVQK  KMLGPSAVEIGNGCFETK  LSGAMDELHNEILELDEK  MLGPSAVEIGNGCFETK  NLNSLSELEVK  SKVIKGSLPLIGEADCLHEK  VIKGSLPLIGEADCLHEK  VSILHEVRPVTSGCFPIMHDR | 39.50% | 96.2  43  62.4  54.5  79.9  53.5  54.9  49  70.3  103  119  67.6  49.7  51.9  57.6 |
| **Hemagglutinin**  **(Q5V9E1_9INFB)** | ADTISSQIELAVLLSDEGIINSEDEHLLALER  AIGNCPIWVK  CLNCTDLDVALGRPK  DRICTGITSSNSPHVVK  GFFGAIAGFLEGGWEGMIAGWHGYTSHGAHGVAVAADLK  GILLPQKVWCASGR  GSLPLIGEADCLHEK  IVVDYMVQK  KMLGPSAVEIGNGCFETK  LSGAMDELHNEILELDEK  MLGPSAVEIGNGCFETK  NLNSLSELEVK  SKVIKGSLPLIGEADCLHEK  VDDLRADTISSQIELAVLLSDEGIINSEDEHLLALER  VIKGSLPLIGEADCLHEK | 36.80% | 96.2  43  62.4  54.5  79.9  53.5  54.9  49  70.3  103  119  67.6  49.7  51.3  51.9 |
| **Matrix protein 1**  **(Q1K9F9_INBAA, Q4G5Z0_9INFB, Q596F7_9INFB, Q596G8_9INFB, Q5V997_9INFB, Q5V999_9INFB, Q5V9A1_9INFB, Q5V9A2_9INFB, Q5V9A5_9INFB, Q5V9A6_9INFB, Q5V9A9_9INFB, Q5V9B1_9INFB, Q5V9B3_9INFB, Q5V9B5_9INFB, Q5V9B7_9INFB, Q5V9B9_9INFB, Q5V9C1_INBMF, Q68IN4_9INFB, Q6F504_9INFB, Q6F506_9INFB, Q6F508_9INFB, Q6F510_9INFB, Q6F512_9INFB, Q6F514_9INFB, Q6F516_9INFB, Q6F518_9INFB, Q6F520_9INFB, Q6F522_9INFB, Q6F524_9INFB, Q6F526_9INFB, Q6F528_9INFB, Q6F530_9INFB, Q6F532_9INFB, Q6F534_9INFB, Q6F536_9INFB, Q6F538_9INFB, Q774Q2_9INFB, Q77ST4_9INFB, Q77SU6_9INFB, Q782K0_9INFB, Q782K1_9INFB, Q784L4_9INFB, Q784L5_9INFB, Q784L6_9INFB, Q80DM2_9INFB, Q9W890_9INFB, Q9WD72_9INFB)** | ALIGASICFLKPK  EFDLDSALEWIK  FITEPLSGMGTTATK  LAEELQSNIGVLR  LGTLCALCEK  RFITEPLSGMGTTATK | 33.90% | 83.9  62.1  84.4  94.7  30.9  59.5 |
| **Membrane protein M1**  **(Q66P73_9INFA)** | AMEQMAGSSEQAAEAMEVASQAR  EPFTEVETYVLSIIPSGPLK  EPFTEVETYVLSIIPSGPLKAEIAQR  GILGFVFTLTVPSER  SIIPSGPLK  TRPILSPLTKGILGFVFTLTVPSER | 29.50% | 75.9  109  82.6  91.2  33.2  42.4 |
| **Nucleoprotein**  **(Q4LCP1_9INFB, Q5V915_9INFB, Q5V916_9INFB, Q5V923_9INFB, Q9WD57_9INFB, Q9WD60_9INFB)** | ALVDQVIGSR  DTAEDYDDLDY  EQVEGMGAALMSIK  LGEFYNQMMVK  MLSMNIEGR  NPGIADIEDLTLLAR  SGGNEVGGDGGSGQISCSPVFAVERPIALSK  TNPIEIPIK  VLSALTGTEFKPR  VVLPISIYAK | 25.40% | 56.2  42.4  56.6  79.3  52.4  68.4  71.7  47.5  75.1  53.9 |
| **Neuraminidase**  **(Q8QHT2_9INFA, Q8QHT3_9INFA)** | IGSKGDVFVIR  TFFLTQGALLNDK  TGSCGPVSSNGANGVKGFSFK  TLMSCPIGEVPSPYNSR  YNGIITDTIK | 16.00% | 49.5  86.5  67.2  71.6  54.8 |
| **Hemagglutinin**  **(Q774L8_9INFA, Q8QT94_9INFA, Q8UYC0_9INFA, Q8V030_9INFA, Q9IGS0_9INFA)** | EIGNGCFEFYHK  EQLSSVSSFER  EVLVLWGIHHPSTSADQQSLYQNADAYVFVGSSR  ITFEATGNLVVPR  MNYYWTLVEPGDK  NLIWLVK | 15.90% | 60  71.9  74.9  102  75.8  38.2 |
|  |  |  |  |
| **Nucleocapsid protein**  **(Q809S3_9INFA)** | EGYSLVGINPFR  FYIQMCTELK  LIQNSITIER  MCSLMQGSTLPR  MVLSAFDER | 15.80% | 92.4  34.6  67.5  92.2  65.7 |
| **Hemagglutinin**  **(Q07FK7_9INFA)** | AYSNCYPYDVPDYASLR  CNSECITPNGSIPNDKPFQNVNR | 7.07% | 61.7  51.6 |
|  |  |  |  |
| **Neuraminidase**  **(Q0A1H1_9INFB)** | LNVETDTAEIR  YGEAYTDTYHSYANK | 5.58% | 58.1  55.4 |
|  |  |  |  |
|  |  |  |  |
|  |  |  |  |

**Table S2.** **TIV, HA and MP1/ NP -specific cytokine responses following vaccination with TIV.**

|  |  | **TIV vaccine** | | |  | **HA peptides** | | |  | **NP + MP peptides** | | |
| --- | --- | --- | --- | --- | --- | --- | --- | --- | --- | --- | --- | --- |
| **Cytokine/ Chemokine** |  | Day 0 | Day 21 | Day 42 |  | Day 0 | Day 21 | Day 42 |  | Day 0 | Day 21 | Day 42 |
| **Proinflammatory** |  |  |  |  |  |  |  |  |  |  |  |  |
| **IFN-γ** |  | 222.92 | 241.85 | 238.64 |  | 5.93 | 75.45 | 22.57 |  | 99.01 | 83.95 | 52.75 |
| **IL-2** |  | 0.01 | 0.01 | 0.01 |  | 0.01 | 0.01 | 0.01 |  | 0.01 | 0.01 | 0.01 |
| **TNF-β (LTα)** |  | 0.01 | 0.01 | 0.01 |  | 0.01 | 0.01 | 0.01 |  | 0.01 | 0.01 | 0.01 |
| **IL-6** |  | 0.14 | 0.01 | 8.38 |  | 895.30 | 1974.06 | 619.06 |  | 127.81 | 43.51 | 131.23 |
| **TNF-α** |  | 73.38 | 162.13 | 113.02 |  | 13.51 | 64.77 | 26.00 |  | 23.56 | 7.81 | 15.51 |
| **MIP-3α (CCL20)** |  | 2.25 | 4.77 | 0.07 |  | 0.01 | 5.13 | 2.62 |  | 0.01 | 0.01 | 0.01 |
| **Th2** |  |  |  |  |  |  |  |  |  |  |  |  |
| **IL-4** |  | 0.04 | 0.03 | 0.04 |  | 0.01 | 0.01 | 0.01 |  | 0.01 | 0.01 | 0.01 |
| **IL-5** |  | 9.85 | 37.74 | 22.87 |  | 0.01 | 0.01 | 0.01 |  | 0.07 | 0.01 | 0.01 |
| **IL-13** |  | 93.94 | 197.74 | 109.50 |  | 11.38 | 11.09 | 8.65 |  | 31.70 | 31.57 | 0.31 |
| **Th17** |  |  |  |  |  |  |  |  |  |  |  |  |
| **IL-17A** |  | 1.51 | 0.09 | 0.01 |  | 0.06 | 0.84 | 0.01 |  | 0.11 | 0.01 | 0.01 |
| **IL-17F** |  | 0.01 | 0.01 | 0.01 |  | 0.01 | 0.08 | 0.01 |  | 0.02 | 0.01 | 0.01 |
| **Th9** |  |  |  |  |  |  |  |  |  |  |  |  |
| **IL-9** |  | 0.01 | 0.01 | 0.01 |  | 0.01 | 0.01 | 0.01 |  | 0.01 | 0.01 | 0.01 |
| **Th22** |  |  |  |  |  |  |  |  |  |  |  |  |
| **IL-22** |  | 0.01 | 0.01 | 0.01 |  | 0.01 | 0.01 | 0.01 |  | 0,01 | 0,01 | 0.01 |
| **Treg** |  |  |  |  |  |  |  |  |  |  |  |  |
| **IL-10** |  | 9.92 | **37.04** | 25.75 |  | 0.85 | 6.90 | 4.53 |  | 3.16 | 7.23 | 8.94 |
| **Tfh** |  |  |  |  |  |  |  |  |  |  |  |  |
| **IL-21** |  | 0.01 | 0.01 | 0.01 |  | 0.01 | 0.01 | 0.01 |  | 0.01 | 0.01 | 0.01 |

Data are median TIV, HA and MP1/ NP -specific cytokine responses, pg/ml, measured by 15-plex assay in supernatants from PBMC cultures that were stimulated with either TIV vaccine or peptide libraries specific for HA and MP1/ NP for 8 days. Boldface and underlined text represents values for which there is statistical evidence of higher median values compared with median values at baseline.

**Table S3.** **Cytokine responses after stimulation with PMA and ionomycin in T-cells isolated from subjects vaccination with TIV.**

|  |  | **PMA** | | |
| --- | --- | --- | --- | --- |
| **Cytokine/ Chemokine** |  | Day 0 | Day 21 | Day 42 |
| **Proinflammatory** |  |  |  |  |
| **IFN-γ** |  | 7950.78 | 8483.79 | 6946.72 |
| **IL-2** |  | 19101.61 | 19266.37 | 18923.44 |
| **TNF-β (LTα)** |  | 0.34 | 0.34 | 0.27 |
| **IL-6** |  | 245.76 | 490.24 | 337.76 |
| **TNF-α** |  | 2617.40 | 2743.53 | 2438.65 |
| **MIP-3α (CCL20)** |  | 242.53 | 254.38 | 168.20 |
| **Th2** |  |  |  |  |
| **IL-4** |  | 0.07 | 0.05 | 0.06 |
| **IL-5** |  | 133.43 | 162.17 | 171.48 |
| **IL-13** |  | 2265.17 | 2868.34 | 2334.75 |
| **Th17** |  |  |  |  |
| **IL-17A** |  | 151.40 | 175.59 | 156.70 |
| **IL-17F** |  | 0.44 | 0.63 | 0.56 |
| **Th9** |  |  |  |  |
| **IL-9** |  | 101.77 | 103.26 | 120.15 |
| **Th22** |  |  |  |  |
| **IL-22** |  | 0.02 | 0.03 | 0.02 |
| **Treg** |  |  |  |  |
| **IL-10** |  | 6.50 | 5.98 | 6.33 |
| **Tfh** |  |  |  |  |
| **IL-21** |  | 11.48 | 11.70 | 15.55 |

Data are median cytokine responses, pg/ml, measured by 15-plex assay in supernatants from PBMC cultures that were stimulated with PMA and ionomycin for 8 days. There was no statistical evidence of different median values between the visits.

**Table S4.** **gH1-specific cytokine responses following vaccination with TIV.**

|  |  | **TIV (n=10)** | | |
| --- | --- | --- | --- | --- |
| **Cytokine/ Chemokine** |  | Day 0 | Day 21 | Day 42 |
| **Proinflammatory** |  |  |  |  |
| **IFN-γ** |  | 59.13 | 120.49 | 82.40 |
| **IL-2** |  | 1.88 | 0.13 | 0.01 |
| **TNF-β (LTα)** |  | 0.01 | 0.01 | 0.01 |
| **IL-6** |  | 4029.77 | 4469.10 | 4294.47 |
| **TNF-α** |  | 293.98 | 259.97 | 349.12 |
| **MIP-3α (CCL20)** |  | 108.47 | 172.23 | 120.95 |
| **Th2** |  |  |  |  |
| **IL-4** |  | 0.01 | 0.01 | 0.01 |
| **IL-5** |  | 2.03 | 2.86 | 4.47 |
| **IL-13** |  | 45.41 | 50.06 | 39.77 |
| **Th17** |  |  |  |  |
| **IL-17A** |  | 2.29 | 5.34 | 3.55 |
| **IL-17F** |  | 0.10 | 0.09 | 0.05 |
| **Th9** |  |  |  |  |
| **IL-9** |  | 0.01 | 0.06 | 0.11 |
| **Th22** |  |  |  |  |
| **IL-22** |  | 0.01 | 0.01 | 0.01 |
| **Treg** |  |  |  |  |
| **IL-10** |  | 21.30 | 24.15 | 15.37 |
| **Tfh** |  |  |  |  |
| **IL-21** |  | 0.01 | 0.01 | 0.01 |

Data are median gH1-specific cytokine responses, pg/ml, measured by 15-plex assay in supernatants from PBMC cultures that were stimulated with peptide libraries specific for gH1 for 8 days. There was no statistical evidence of different median values between the visits.

**Table S5.** **Qbeta-specific cytokine responses following vaccination with gH1-Qbeta.**

|  |  |  | **Non-adjuvanted** |  |  |  | **Adjuvanted** |  |
| --- | --- | --- | --- | --- | --- | --- | --- | --- |
| **Cytokine/ Chemokine** |  | Day 0 | Day 21 | Day 42 |  | Day 0 | Day 21 | Day 42 |
| **Proinflammatory** |  |  |  |  |  |  |  |  |
| **IFN-γ** |  | 1.97 | **8.73** | **11.90** |  | 1.06 | **63.70** | **80.77** |
| **IL-2** |  | 5.73 | 0.39 | 0.97 |  | 3.88 | 0.01 | 0.01 |
| **TNF-β (LTα)** |  | 0.01 | 0.01 | 0.01 |  | 0.01 | 0.01 | 0.01 |
| **IL-6** |  | 2851.67 | 3101.27 | 2144.62 |  | 2597.47 | 2987.51 | 3079.99 |
| **TNF-α** |  | 12.34 | 16.83 | 25.85 |  | 25.03 | 37.74 | 37.37 |
| **MIP-3α (CCL20)** |  | 71.01 | 91.97 | 49.30 |  | 54.18 | 50.43 | 40.06 |
| **Th2** |  |  |  |  |  |  |  |  |
| **IL-4** |  | 0.01 | 0.01 | 0.01 |  | 0.01 | 0.01 | 0.01 |
| **IL-5** |  | 0.01 | 2.59 | **1.93** |  | 0.01 | **2.29** | **2.50** |
| **IL-13** |  | 6.46 | 16.15 | 18.54 |  | 6.89 | **25.49** | **37.93** |
| **Th17** |  |  |  |  |  |  |  |  |
| **IL-17A** |  | 0.01 | **4.43** | **5.27** |  | 0.01 | **11.28** | **5.68** |
| **IL-17F** |  | 0.01 | **0.06** | **0.06** |  | 0.01 | **0.17** | **0.15** |
| **Th9** |  |  |  |  |  |  |  |  |
| **IL-9** |  | 0.01 | 0.01 | 0.01 |  | 0.01 | 0.34 | **1.46** |
| **Th22** |  |  |  |  |  |  |  |  |
| **IL-22** |  | 0.01 | 0.01 | 0.01 |  | 0.01 | 0.01 | 0.01 |
| **Treg** |  |  |  |  |  |  |  |  |
| **IL-10** |  | 7.80 | 8.58 | 9.44 |  | 5.85 | 8.26 | 9.68 |
| **Tfh** |  |  |  |  |  |  |  |  |
| **IL-21** |  | 0.01 | 0.01 | 0.01 |  | 0.01 | 0.01 | **0.01** |

Data are median Qbeta-specific cytokine responses, pg/ml, measured by 15-plex assay in supernatants from PBMC cultures that were stimulated with peptide libraries specific for Qbeta for 8 days. Boldface and underlined text represents values for which there is statistical evidence of higher median values compared with median values at baseline. In the non-adjuvanted group between one and two doses of vaccine statistically lower median values were observed for MIP-3α.

**Table S6.** **PepMix™ Influenza A (HA /California (H1N1)).**

| **Peptide #** | **Peptide sequence** |
| --- | --- |
| 1 | MKAILVVLLYTFATA |
| 2 | LVVLLYTFATANADT |
| 3 | LYTFATANADTLCIG |
| 4 | ATANADTLCIGYHAN |
| 5 | ADTLCIGYHANNSTD |
| 6 | CIGYHANNSTDTVDT |
| 7 | HANNSTDTVDTVLEK |
| 8 | STDTVDTVLEKNVTV |
| 9 | VDTVLEKNVTVTHSV |
| 10 | LEKNVTVTHSVNLLE |
| 11 | VTVTHSVNLLEDKHN |
| 12 | HSVNLLEDKHNGKLC |
| 13 | LLEDKHNGKLCKLRG |
| 14 | KHNGKLCKLRGVAPL |
| 15 | KLCKLRGVAPLHLGK |
| 16 | LRGVAPLHLGKCNIA |
| 17 | APLHLGKCNIAGWIL |
| 18 | LGKCNIAGWILGNPE |
| 19 | NIAGWILGNPECESL |
| 20 | WILGNPECESLSTAS |
| 21 | NPECESLSTASSWSY |
| 22 | ESLSTASSWSYIVET |
| 23 | TASSWSYIVETPSSD |
| 24 | WSYIVETPSSDNGTC |
| 25 | VETPSSDNGTCYPGD |
| 26 | SSDNGTCYPGDFIDY |
| 27 | GTCYPGDFIDYEELR |
| 28 | PGDFIDYEELREQLS |
| 29 | IDYEELREQLSSVSS |
| 30 | ELREQLSSVSSFERF |
| 31 | QLSSVSSFERFEIFP |
| 32 | VSSFERFEIFPKTSS |
| 33 | ERFEIFPKTSSWPNH |
| 34 | IFPKTSSWPNHDSNK |
| 35 | TSSWPNHDSNKGVTA |
| 36 | PNHDSNKGVTAACPH |
| 37 | SNKGVTAACPHAGAK |
| 38 | VTAACPHAGAKSFYK |
| 39 | CPHAGAKSFYKNLIW |
| 40 | GAKSFYKNLIWLVKK |
| 41 | FYKNLIWLVKKGNSY |
| 42 | LIWLVKKGNSYPKLS |
| 43 | VKKGNSYPKLSKSYI |
| 44 | NSYPKLSKSYINDKG |
| 45 | KLSKSYINDKGKEVL |
| 46 | SYINDKGKEVLVLWG |
| 47 | DKGKEVLVLWGIHHP |
| 48 | EVLVLWGIHHPSTSA |
| 49 | LWGIHHPSTSADQQS |
| 50 | HHPSTSADQQSLYQN |
| 51 | TSADQQSLYQNADAY |
| 52 | QQSLYQNADAYVFVG |
| 53 | YQNADAYVFVGSSRY |
| 54 | DAYVFVGSSRYSKKF |
| 55 | FVGSSRYSKKFKPEI |
| 56 | SRYSKKFKPEIAIRP |
| 57 | KKFKPEIAIRPKVRD |
| 58 | PEIAIRPKVRDQEGR |
| 59 | IRPKVRDQEGRMNYY |
| 60 | VRDQEGRMNYYWTLV |
| 61 | EGRMNYYWTLVEPGD |
| 62 | NYYWTLVEPGDKITF |
| 63 | TLVEPGDKITFEATG |
| 64 | PGDKITFEATGNLVV |
| 65 | ITFEATGNLVVPRYA |
| 66 | ATGNLVVPRYAFAME |
| 67 | LVVPRYAFAMERNAG |
| 68 | RYAFAMERNAGSGII |
| 69 | AMERNAGSGIIISDT |
| 70 | NAGSGIIISDTPVHD |
| 71 | GIIISDTPVHDCNTT |
| 72 | SDTPVHDCNTTCQTP |
| 73 | VHDCNTTCQTPKGAI |
| 74 | NTTCQTPKGAINTSL |
| 75 | QTPKGAINTSLPFQN |
| 76 | GAINTSLPFQNIHPI |
| 77 | TSLPFQNIHPITIGK |
| 78 | FQNIHPITIGKCPKY |
| 79 | HPITIGKCPKYVKST |
| 80 | IGKCPKYVKSTKLRL |
| 81 | PKYVKSTKLRLATGL |
| 82 | KSTKLRLATGLRNIP |
| 83 | LRLATGLRNIPSIQS |
| 84 | TGLRNIPSIQSRGLF |
| 85 | NIPSIQSRGLFGAIA |
| 86 | IQSRGLFGAIAGFIE |
| 87 | GLFGAIAGFIEGGWT |
| 88 | AIAGFIEGGWTGMVD |
| 89 | FIEGGWTGMVDGWYG |
| 90 | GWTGMVDGWYGYHHQ |
| 91 | MVDGWYGYHHQNEQG |
| 92 | WYGYHHQNEQGSGYA |
| 93 | HHQNEQGSGYAADLK |
| 94 | EQGSGYAADLKSTQN |
| 95 | GYAADLKSTQNAIDE |
| 96 | DLKSTQNAIDEITNK |
| 97 | TQNAIDEITNKVNSV |
| 98 | IDEITNKVNSVIEKM |
| 99 | TNKVNSVIEKMNTQF |
| 100 | NSVIEKMNTQFTAVG |
| 101 | EKMNTQFTAVGKEFN |
| 102 | TQFTAVGKEFNHLEK |
| 103 | AVGKEFNHLEKRIEN |
| 104 | EFNHLEKRIENLNKK |
| 105 | LEKRIENLNKKVDDG |
| 106 | IENLNKKVDDGFLDI |
| 107 | NKKVDDGFLDIWTYN |
| 108 | DDGFLDIWTYNAELL |
| 109 | LDIWTYNAELLVLLE |
| 110 | TYNAELLVLLENERT |
| 111 | ELLVLLENERTLDYH |
| 112 | LLENERTLDYHDSNV |
| 113 | ERTLDYHDSNVKNLY |
| 114 | DYHDSNVKNLYEKVR |
| 115 | SNVKNLYEKVRSQLK |
| 116 | NLYEKVRSQLKNNAK |
| 117 | KVRSQLKNNAKEIGN |
| 118 | QLKNNAKEIGNGCFE |
| 119 | NAKEIGNGCFEFYHK |
| 120 | IGNGCFEFYHKCDNT |
| 121 | CFEFYHKCDNTCMES |
| 122 | YHKCDNTCMESVKNG |
| 123 | DNTCMESVKNGTYDY |
| 124 | MESVKNGTYDYPKYS |
| 125 | KNGTYDYPKYSEEAK |
| 126 | YDYPKYSEEAKLNRE |
| 127 | KYSEEAKLNREEIDG |
| 128 | EAKLNREEIDGVKLE |
| 129 | NREEIDGVKLESTRI |
| 130 | IDGVKLESTRIYQIL |
| 131 | KLESTRIYQILAIYS |
| 132 | TRIYQILAIYSTVAS |
| 133 | QILAIYSTVASSLVL |
| 134 | IYSTVASSLVLVVSL |
| 135 | VASSLVLVVSLGAIS |
| 136 | LVLVVSLGAISFWMC |
| 137 | VSLGAISFWMCSNGS |
| 138 | AISFWMCSNGSLQCR |
| 139 | WMCSNGSLQCRICI |

Pool of 139 peptides derived from a peptide scan (15mers with 11 aa overlap) through Hemagglutinin (Swiss-Prot ID: C3W5X2) of Influenza A virus (A/California/07/2009(H1N1)) for T cell assays.

**Table S7.** **PepMix™ Influenza A (MP1 /California (H1N1)).**

| **Peptide #** | **Peptide sequence** |
| --- | --- |
| 1 | MSLLTEVETYVLSII |
| 2 | TEVETYVLSIIPSGP |
| 3 | TYVLSIIPSGPLKAE |
| 4 | SIIPSGPLKAEIAQR |
| 5 | SGPLKAEIAQRLESV |
| 6 | KAEIAQRLESVFAGK |
| 7 | AQRLESVFAGKNTDL |
| 8 | ESVFAGKNTDLEALM |
| 9 | AGKNTDLEALMEWLK |
| 10 | TDLEALMEWLKTRPI |
| 11 | ALMEWLKTRPILSPL |
| 12 | WLKTRPILSPLTKGI |
| 13 | RPILSPLTKGILGFV |
| 14 | SPLTKGILGFVFTLT |
| 15 | KGILGFVFTLTVPSE |
| 16 | GFVFTLTVPSERGLQ |
| 17 | TLTVPSERGLQRRRF |
| 18 | PSERGLQRRRFVQNA |
| 19 | GLQRRRFVQNALNGN |
| 20 | RRFVQNALNGNGDPN |
| 21 | QNALNGNGDPNNMDR |
| 22 | NGNGDPNNMDRAVKL |
| 23 | DPNNMDRAVKLYKKL |
| 24 | MDRAVKLYKKLKREI |
| 25 | VKLYKKLKREITFHG |
| 26 | KKLKREITFHGAKEV |
| 27 | REITFHGAKEVSLSY |
| 28 | FHGAKEVSLSYSTGA |
| 29 | KEVSLSYSTGALASC |
| 30 | LSYSTGALASCMGLI |
| 31 | TGALASCMGLIYNRM |
| 32 | ASCMGLIYNRMGTVT |
| 33 | GLIYNRMGTVTTEAA |
| 34 | NRMGTVTTEAAFGLV |
| 35 | TVTTEAAFGLVCATC |
| 36 | EAAFGLVCATCEQIA |
| 37 | GLVCATCEQIADSQH |
| 38 | ATCEQIADSQHRSHR |
| 39 | QIADSQHRSHRQMAT |
| 40 | SQHRSHRQMATTTNP |
| 41 | SHRQMATTTNPLIRH |
| 42 | MATTTNPLIRHENRM |
| 43 | TNPLIRHENRMVLAS |
| 44 | IRHENRMVLASTTAK |
| 45 | NRMVLASTTAKAMEQ |
| 46 | LASTTAKAMEQMAGS |
| 47 | TAKAMEQMAGSSEQA |
| 48 | MEQMAGSSEQAAEAM |
| 49 | AGSSEQAAEAMEVAN |
| 50 | EQAAEAMEVANQTRQ |
| 51 | EAMEVANQTRQMVHA |
| 52 | VANQTRQMVHAMRTI |
| 53 | TRQMVHAMRTIGTHP |
| 54 | VHAMRTIGTHPSSSA |
| 55 | RTIGTHPSSSAGLKD |
| 56 | THPSSSAGLKDDLLE |
| 57 | SSAGLKDDLLENLQA |
| 58 | LKDDLLENLQAYQKR |
| 59 | LLENLQAYQKRMGVQ |
| 60 | LQAYQKRMGVQMQRF |
| 61 | QKRMGVQMQRFK |

Pool of 61 peptides derived from a peptide scan through Matrix protein 1 of Influenza A virus (A/California/08/2009(H1N1)) for T cell assays.

**Table S8.** **PepMix™ Influenza A (Neuroamidase /California (H1N1).**

| **Peptide #** | **Peptide sequence** |
| --- | --- |
| 1 | MNPNQKIITIGSVCM |
| 2 | QKIITIGSVCMTIGM |
| 3 | TIGSVCMTIGMANLI |
| 4 | VCMTIGMANLILQIG |
| 5 | IGMANLILQIGNIIS |
| 6 | NLILQIGNIISIWIS |
| 7 | QIGNIISIWISHSIQ |
| 8 | IISIWISHSIQLGNQ |
| 9 | WISHSIQLGNQNQIE |
| 10 | SIQLGNQNQIETCNQ |
| 11 | GNQNQIETCNQSVIT |
| 12 | QIETCNQSVITYENN |
| 13 | CNQSVITYENNTWVN |
| 14 | VITYENNTWVNQTYV |
| 15 | ENNTWVNQTYVNISN |
| 16 | WVNQTYVNISNTNFA |
| 17 | TYVNISNTNFAAGQS |
| 18 | ISNTNFAAGQSVVSV |
| 19 | NFAAGQSVVSVKLAG |
| 20 | GQSVVSVKLAGNSSL |
| 21 | VSVKLAGNSSLCPVS |
| 22 | LAGNSSLCPVSGWAI |
| 23 | SSLCPVSGWAIYSKD |
| 24 | PVSGWAIYSKDNSVR |
| 25 | WAIYSKDNSVRIGSK |
| 26 | SKDNSVRIGSKGDVF |
| 27 | SVRIGSKGDVFVIRE |
| 28 | GSKGDVFVIREPFIS |
| 29 | DVFVIREPFISCSPL |
| 30 | IREPFISCSPLECRT |
| 31 | FISCSPLECRTFFLT |
| 32 | SPLECRTFFLTQGAL |
| 33 | CRTFFLTQGALLNDK |
| 34 | FLTQGALLNDKHSNG |
| 35 | GALLNDKHSNGTIKD |
| 36 | NDKHSNGTIKDRSPY |
| 37 | SNGTIKDRSPYRTLM |
| 38 | IKDRSPYRTLMSCPI |
| 39 | SPYRTLMSCPIGEVP |
| 40 | TLMSCPIGEVPSPYN |
| 41 | CPIGEVPSPYNSRFE |
| 42 | EVPSPYNSRFESVAW |
| 43 | PYNSRFESVAWSASA |
| 44 | RFESVAWSASACHDG |
| 45 | VAWSASACHDGINWL |
| 46 | ASACHDGINWLTIGI |
| 47 | HDGINWLTIGISGPD |
| 48 | NWLTIGISGPDNGAV |
| 49 | IGISGPDNGAVAVLK |
| 50 | GPDNGAVAVLKYNGI |
| 51 | GAVAVLKYNGIITDT |
| 52 | VLKYNGIITDTIKSW |
| 53 | NGIITDTIKSWRNNI |
| 54 | TDTIKSWRNNILRTQ |
| 55 | KSWRNNILRTQESEC |
| 56 | NNILRTQESECACVN |
| 57 | RTQESECACVNGSCF |
| 58 | SECACVNGSCFTVMT |
| 59 | CVNGSCFTVMTDGPS |
| 60 | SCFTVMTDGPSNGQA |
| 61 | VMTDGPSNGQASYKI |
| 62 | GPSNGQASYKIFRIE |
| 63 | GQASYKIFRIEKGKI |
| 64 | YKIFRIEKGKIVKSV |
| 65 | RIEKGKIVKSVEMNA |
| 66 | GKIVKSVEMNAPNYH |
| 67 | KSVEMNAPNYHYEEC |
| 68 | MNAPNYHYEECSCYP |
| 69 | NYHYEECSCYPDSSE |
| 70 | EECSCYPDSSEITCV |
| 71 | CYPDSSEITCVCRDN |
| 72 | SSEITCVCRDNWHGS |
| 73 | TCVCRDNWHGSNRPW |
| 74 | RDNWHGSNRPWVSFN |
| 75 | HGSNRPWVSFNQNLE |
| 76 | RPWVSFNQNLEYQIG |
| 77 | SFNQNLEYQIGYICS |
| 78 | NLEYQIGYICSGIFG |
| 79 | QIGYICSGIFGDNPR |
| 80 | ICSGIFGDNPRPNDK |
| 81 | IFGDNPRPNDKTGSC |
| 82 | NPRPNDKTGSCGPVS |
| 83 | NDKTGSCGPVSSNGA |
| 84 | GSCGPVSSNGANGVK |
| 85 | PVSSNGANGVKGFSF |
| 86 | NGANGVKGFSFKYGN |
| 87 | GVKGFSFKYGNGVWI |
| 88 | FSFKYGNGVWIGRTK |
| 89 | YGNGVWIGRTKSISS |
| 90 | VWIGRTKSISSRNGF |
| 91 | RTKSISSRNGFEMIW |
| 92 | ISSRNGFEMIWDPNG |
| 93 | NGFEMIWDPNGWTGT |
| 94 | MIWDPNGWTGTDNNF |
| 95 | PNGWTGTDNNFSIKQ |
| 96 | TGTDNNFSIKQDIVG |
| 97 | NNFSIKQDIVGINEW |
| 98 | IKQDIVGINEWSGYS |
| 99 | IVGINEWSGYSGSFV |
| 100 | NEWSGYSGSFVQHPE |
| 101 | GYSGSFVQHPELTGL |
| 102 | SFVQHPELTGLDCIR |
| 103 | HPELTGLDCIRPCFW |
| 104 | TGLDCIRPCFWVELI |
| 105 | CIRPCFWVELIRGRP |
| 106 | CFWVELIRGRPKENT |
| 107 | ELIRGRPKENTIWTS |
| 108 | GRPKENTIWTSGSSI |
| 109 | ENTIWTSGSSISFCG |
| 110 | WTSGSSISFCGVNSD |
| 111 | SSISFCGVNSDTVGW |
| 112 | FCGVNSDTVGWSWPD |
| 113 | NSDTVGWSWPDGAEL |
| 114 | VGWSWPDGAELPFTI |
| 115 | WPDGAELPFTIDK |

Pool of 115 peptides derived from a peptide scan through Neuraminidase of Influenza A virus (A/California/08/2009(H1N1)) for T cell assays.

**Table S9.** **Influenza A (California (H1N1)) gH1 peptide sequences.**

| **Peptide #** | **Peptide sequence** |
| --- | --- |
| 1 | MLLEDKHNGKLCKLR |
| 2 | DKHNGKLCKLRGVAP |
| 3 | GKLCKLRGVAPLHLG |
| 4 | KLRGVAPLHLGKCNI |
| 5 | VAPLHLGKCNIAGWI |
| 6 | HLGKCNIAGWILGNP |
| 7 | CNIAGWILGNPECES |
| 8 | GWILGNPECESLSTA |
| 9 | GNPECESLSTASSWS |
| 10 | CESLSTASSWSYIVE |
| 11 | STASSWSYIVETPSS |
| 12 | SWSYIVETPSSDNGT |
| 13 | IVETPSSDNGTCYPG |
| 14 | PSSDNGTCYPGDFID |
| 15 | NGTCYPGDFIDYEEL |
| 16 | YPGDFIDYEELREQL |
| 17 | FIDYEELREQLSSVS |
| 18 | EELREQLSSVSSFER |
| 19 | EQLSSVSSFERFEIF |
| 20 | SVSSFERFEIFPKTS |
| 21 | FERFEIFPKTSSWPN |
| 22 | EIFPKTSSWPNHDSN |
| 23 | KTSSWPNHDSNKGVT |
| 24 | WPNHDSNKGVTAACP |
| 25 | DSNKGVTAACPHAGA |
| 26 | GVTAACPHAGAKSFY |
| 27 | ACPHAGAKSFYKNLI |
| 28 | AGAKSFYKNLIWLVK |
| 29 | SFYKNLIWLVKKGNS |
| 30 | NLIWLVKKGNSYPKL |
| 31 | LVKKGNSYPKLSKSY |
| 32 | GNSYPKLSKSYINDK |
| 33 | PKLSKSYINDKGKEV |
| 34 | KSYINDKGKEVLVLW |
| 35 | NDKGKEVLVLWGIHH |
| 36 | KEVLVLWGIHHPSTS |
| 37 | VLWGIHHPSTSADQQ |
| 38 | IHHPSTSADQQSLYQ |
| 39 | STSADQQSLYQNADA |
| 40 | DQQSLYQNADAYVFV |
| 41 | LYQNADAYVFVGSSR |
| 42 | ADAYVFVGSSRYSKK |
| 43 | VFVGSSRYSKKFKPE |
| 44 | SSRYSKKFKPEIAIR |
| 45 | SKKFKPEIAIRPKVR |
| 46 | KPEIAIRPKVRDREG |
| 47 | AIRPKVRDREGRMNY |
| 48 | KVRDREGRMNYYWTL |
| 49 | REGRMNYYWTLVEPG |
| 50 | MNYYWTLVEPGDKIT |
| 51 | WTLVEPGDKITFEAT |
| 52 | EPGDKITFEATGNLV |
| 53 | KITFEATGNLVVPRY |
| 54 | EATGNLVVPRYAFAM |
| 55 | NLVVPRYAFAMERNA |
| 56 | PRYAFAMERNAGSGI |
| 57 | FAMERNAGSGIIISD |
| 58 | RNAGSGIIISDTPVH |
| 59 | SGIIISDTPVHDCNT |
| 60 | ISDTPVHDCNTTCQT |
| 61 | PVHDCNTTCQTPKGA |
| 62 | CNTTCQTPKGAINTS |
| 63 | CQTPKGAINTSLPFQ |
| 64 | KGAINTSLPFQNIHP |
| 65 | NTSLPFQNIHPITIG |
| 66 | PFQNIHPITIGKCPK |
| 67 | IHPITIGKCPKYVKG |
| 68 | TIGKCPKYVKGGCG |

**Table S10.** **Qbeta peptide sequences.**

| **Peptide #** | **Peptide sequence** |
| --- | --- |
| 1 | AKLETVTLGNIGKDG |
| 2 | TVTLGNIGKDGKQTL |
| 3 | GNIGKDGKQTLVLNP |
| 4 | KDGKQTLVLNPRGVN |
| 5 | QTLVLNPRGVNPTNG |
| 6 | LNPRGVNPTNGVASL |
| 7 | GVNPTNGVASLSQAG |
| 8 | TNGVASLSQAGAVPA |
| 9 | ASLSQAGAVPALEKR |
| 10 | QAGAVPALEKRVTVS |
| 11 | VPALEKRVTVSVSQP |
| 12 | EKRVTVSVSQPSRNR |
| 13 | TVSVSQPSRNRKNYK |
| 14 | SQPSRNRKNYKVQVK |
| 15 | RNRKNYKVQVKIQNP |
| 16 | NYKVQVKIQNPTACT |
| 17 | QVKIQNPTACTANGS |
| 18 | QNPTACTANGSCDPS |
| 19 | ACTANGSCDPSVTRQ |
| 20 | NGSCDPSVTRQAYAD |
| 21 | DPSVTRQAYADVTFS |
| 22 | TRQAYADVTFSFTQY |
| 23 | YADVTFSFTQYSTDE |
| 24 | TFSFTQYSTDEERAF |
| 25 | TQYSTDEERAFVRTE |
| 26 | TDEERAFVRTELAAL |
| 27 | RAFVRTELAALLASP |
| 28 | RTELAALLASPLLID |
| 29 | AALLASPLLIDAIDQ |
| 30 | ASPLLIDAIDQLNPAY |
